# Disease-relevant mutations alter amino acid co-evolution networks in the second nucleotide binding domain of CFTR

**DOI:** 10.1371/journal.pone.0227668

**Published:** 2020-01-24

**Authors:** Gabrianne Ivey, Robert T. Youker

**Affiliations:** 1 Kyder Christian Academy, Franklin, North Carolina, United States of America; 2 Southwestern Community College, Sylva, North Carolina, United States of America; 3 Department of Biology, Western Carolina University, Cullowhee, North Carolina, United States of America; Odense University Hospital, DENMARK

## Abstract

Cystic Fibrosis (CF) is an inherited disease caused by mutations in the cystic fibrosis transmembrane conductance regulator (CFTR) ion channel. Mutations in CFTR cause impaired chloride ion transport in the epithelial tissues of patients leading to cardiopulmonary decline and pancreatic insufficiency in the most severely affected patients. CFTR is composed of twelve membrane-spanning domains, two nucleotide-binding domains (NBDs), and a regulatory domain. The most common mutation in CFTR is a deletion of phenylalanine at position 508 (ΔF508) in NBD1. Previous research has primarily concentrated on the structure and dynamics of the NBD1 domain; However numerous pathological mutations have also been found in the lesser-studied NBD2 domain. We have investigated the amino acid co-evolved network of interactions in NBD2, and the changes that occur in that network upon the introduction of CF and CF-related mutations (S1251N(T), S1235R, D1270N, N1303K(T)). Extensive coupling between the α- and β-subdomains were identified with residues in, or near Walker A, Walker B, H-loop and C-loop motifs. Alterations in the predicted residue network varied from moderate for the S1251T perturbation to more severe for N1303T. The S1235R and D1270N networks varied greatly compared to the wildtype, but these CF mutations only affect ion transport preference and do not severely disrupt CFTR function, suggesting dynamic flexibility in the network of interactions in NBD2. Our results also suggest that inappropriate interactions between the β-subdomain and Q-loop could be detrimental. We also identified mutations predicted to stabilize the NBD2 residue network upon introduction of the CF and CF-related mutations, and these predicted mutations are scored as benign by the MUTPRED2 algorithm. Our results suggest the level of disruption of the co-evolution predictions of the amino acid networks in NBD2 does not have a straightforward correlation with the severity of the CF phenotypes observed.

## Introduction

Cystic fibrosis (CF) is one of the most common autosomal recessive genetic disorders among Caucasians and results from a mutation in the cystic fibrosis transmembrane conductance regulator (CFTR) [1,2]. CFTR is an integral chloride channel that regulates ion and water secretion, and it is expressed predominantly in epithelial tissues. Clinical manifestations of CF include cardio-pulmonary abnormalities, diabetes mellitus, male infertility and pancreatic insufficiency [3]. CFTR is composed of two transmembrane domains (TMDs), two nucleotide-binding domains (NBDs), and a regulatory domain (R). Phosphorylation of the R domain and ATP-dependent “head-to-tail” heterodimerization of the NBDs controls ion channel gating [4,5]. The most common mutation in CF patients is a deletion of a single phenylalanine at position 508 in NBD1 (ΔF508) [6]. Thus, numerous studies have investigated NBD1’s structure and function, and its change in response to mutation [7–10]. However, disease-causing mutations also occur in NBD2 of CFTR and several mutations, such as S1235R and N1303K constitute a significant prevalence in CF patients [11,12]. The NBDs of CFTR are composed of α-helical and β-sheet subdomains that contain important motifs (Walker A, Walker B, C-loop, Q-loop, H-loop) required for proper ATP binding and hydrolysis [13–15]. Interestingly, the Regulatory Extension (RE) and Regulatory Insertion (RI) regions, which are unique to CFTR and are considered crucial for NBD1 dynamics, are missing from NBD2 [7]. As opposed to ΔF508, the S1235R and N1303K mutants are not rescued by low temperature [16]. This suggests NBD2 dynamics are not necessarily identical to NBD1, but less is known about the allosteric control of NBD2.

Because it is difficult to investigate the allosteric control of NBD1 and NBD2 *in vitro* using biochemical methods, there is a reliance on computational methods to uncover the dynamics of these NBDs [7, 17–20]. Computational methods, such as covariance, or co-evolution algorithms, have been employed to probe the dynamics of NBD1 [17]. Covariance algorithms have been used to identify co-evolved amino acids (a.k.a residues) in a variety of proteins including serine/threonine kinases, ABC transporters, G-protein-coupled receptors, proteases, SH3 domains and PDZ domains [18, 19, 21–25]. These statistical methods have been widely used to predict amino acid interactions as they have the potential to not only find physically linked positions that would also be identified by x-ray crystallography, but also hidden coupling, whereby the amino acids interact due to protein allostery [22]. Co-evolution algorithms are a bioinformatic tool used to measure the covariation between pairs of amino acids in an MSA with the increasing coupling score interpreted as increased evolutionary dependency between residues. This method, then, is a “higher order” evaluation of MSAs than the method of identifying conservation of a single residue. Because it is statistically more rigorous, it requires a larger number of homologous sequences. The ATP-binding cassette (ABC) transporter superfamily is amenable to covariance analysis because it is one of the oldest and most diverse group of proteins [26]. CFTR is a member of the ABC transporter family (ABC, subfamily C, member 7) and coupling analysis has been performed on CFTR, or concentrating on the NBD1 domain, identified key residues needed for folding and stability [17, 19]. Szollosi and colleagues used statistical coupling analysis to determine potential coupling in NBD2 of CFTR and they identified three coupled positions: L1303-I1296-R1358 [18]. However, the effects of disease-causing, or associated mutations on the predicted NBD2 co-evolved network of residues is not known.

We sought to confirm previously-identified coupled residues in NBD2 and, more importantly, to identify any potential changes in the predicted co-evolved network in response to disease-causing, or associated mutations. We chose to focus on four mutations (N1303K, S1235R, S1251N, and D1270N) based on prevalence in CF population, and varying clinical outcomes [27–32]. The N1303K mutation is the most frequent CF-causing mutation in Europe (7.8% in southwestern France), and patients have severe clinical outcomes such as pancreatitis, pulmonary complications, and diabetes mellitus [11,27]. The S1251N mutation is located in the Walker A motif of NBD2 and is the most common class III-IV mutation (gating/conductance defect) in the Netherlands with an 1.2% occurrence [32]. This mutation alters channel gating resulting in less than 10% channel function [33,34]. The S1235R and D1270N mutations are non-CF causing but affected individuals present with pancreatitis, increased risk for rhinosinusitis, and male infertility [29]. The S1235R mutation occurs with a relative allele frequency of approximately 0.1% that is significantly greater than other rare CF mutations [6].

We used five independent co-evolutionary algorithms for predicting amino acid co-evolution in NBD2: Explicit Likelihood of Subset Covariance (ELSC), Observed Minus Expected Squared (OMES), McLachan Substitution Correlation Matrix (McBASC), Statistical Coupling Analysis (SCA), and the Blocks In Sequences (BIS)[35–39]. Based on these computational analyses, we identified coupled positions in the wild type NBD2 of CFTR and determined changes in predicted coupled position for CF-causing and CF-associated mutations. Predicted interactions between the α- and β-subdomains of NBD2 were disrupted to varying degrees depending on the mutation investigated. In addition to identifying changes in residue interactions, we also identified potential thermo-stabilizing mutations using statistical means as described by Sullivan and Durani [40,41]. The stabilizing mutations we identified were in close proximity to predicted coupled residues that were identified in key functional motifs (Walker A and Walker B, C-loop, Q-loop and H-loop.). Finally, these predicted stabilizing mutations were scored as pathogenic, or benign according to the MUTPRED2 algorithm.

## Materials and methods

### Co-evolution analysis methods

Four independent statistical methods were used to determine covariance in NBD2 of CFTR. The version of the covariance analyses used were written by A. Fodor et al. in Java [35,42].

#### Observed Minus Expected Squared (OMES)

The Observed Minus Expected Squared covariance algorithm, which was originally created by Kass and Horovitz, produces a list, termed *L*, of each possible pair of amino acids at positions *i* and *j* in a given multiple sequence alignment (MSA)[36]. If either position *i* or *j* contains a gap, that amino acid pair is not recorded. The equation is described as:

$$\sum_{1}^{L}\frac{{\left(N_{obs}-N_{ex}\right)}^{2}}{N_{valid}}$$

where *N*_*obs*_ is the number of occurrences of a specific amino acid pair, *N*_*valid*_ is the total number of residue pairs in the MSA that do not contain gaps at either position *i* or *j*, and *N*_*ex*_ is the number of expected occurrences of a specific pair calculated by the following equation:

$$N_{ex}=\frac{C_{xi}C_{yi}}{N_{valid}}$$

*C*_xi_ is the probability that residue *i* will be at position x, and *C*_yi_ is the probability that residue *j* will be at position y. *N*_*ex*_ is calculated for all possible pairs of residues (*L*) and then summed to give the covariance score for that coupled position.

#### McLachlan Based Substitution Correlation (McBASC)

This covariance analysis is based on Gobel *et al*. algorithm, and determines which residues are allowable at a given position by substituting all possible amino acids in that position [37]. For every position *i*, an *N*x*N* array is created with *N* being the number of sequences in the MSA, and one dimension running from *k = 1* to *N*, and the other from *l = 1* to *N*. Each element contains two amino acids, one corresponding to *k* and the other to *l*. A score, termed *S*_*ikl*_, is given for the substitution of *k* and *l* based on the similarity of the residues where a high score refers to a high conservation, and low score implies low conservation. If a gap is at either *k* or *l*, it is given the score of 0. This scoring matrix is applied throughout the *N*x*N* array, and the average score, S_i_, is calculated. The mutational behavior at position *i* and *j* is then calculated by the following equation:

$$r_{i,j}=\frac{1}{N^{2}}\frac{\sum_{kl}\left(S_{ikl}-\langle S_{i}\rangle )-(S_{jkl}-\langle S_{j}\rangle \right)}{\sigma_{i}\sigma_{j}}$$

where σ_i_ σ_j_ is the standard deviation of the array generated from position *i* and *j* respectively, *S*_*jkl*_ is the similarity between amino acid at *k* and *l* in column *j*, and *S*_*j*_ is the average score for position *j*. The absolute values of scores were used and columns that were originally given a value of 2 were changed to 0 as they indicated conserved or gapped columns.

#### Statistical Coupling Analysis (SCA)

This coupling analysis technique was initially developed by Lockless and Ranganathan [38] and is a perturbation-based method that calculates the change in coupling scores in a full MSA compared to a sub-alignment [38]. Each column is given a value according to

$${\Delta G}_{i}=\sqrt{\sum_{x}{\left(ln\frac{P_{i}^{x}}{P_{MSA}^{x}}\right)}^{2}}$$

In the equation above, *P*_*i*_^*x*^ is the probability of amino acid *x* occurring at position *i*, while *P*^*x*^ is the probability of position *x* in the entire MSA. The covariance is determined by

$$\Delta \Delta G_{i,j}=\sqrt{\sum_{x}{(\ln\frac{P_{i|\&j}^{x}}{P_{MSA|\&j}^{x}}-\ln\frac{P_{i}^{x}}{P_{MSA}^{x}})}^{2}}$$

*P*^*x*^_*i|δj*_ divided by *P*^*x*^_*MSA|δj*_ is the probability of position *i* containing amino acid x after a sub- alignment is created where position *j* only contains sequences with a specific amino acid, and *P*^*x*^_*i*_ divided by *P*^*x*^_*MSA*_ is the probability of finding that same amino acid at position *i* with all the sequences in the MSA. To determine the ΔΔ*G*_i,j_, the calculation is repeated for all 20 amino acids and the score is summed. To avoid a high correlation score for columns that contain primarily gaps, positions with a frequency of gaps greater than 0.2 were removed.

#### Explicit Likelihood of Subset Covariance (ELSC)

The explicit likelihood of subset covariance analysis, (ELSC), was created by Dekker *et al*. [35] and is a perturbation-based analysis where the effect on position *j*, when taking the sequence that contains only amino acid x at positions *i*, is assessed by first determining the amino acid composition at position *j* in the sub-alignment. The sub-alignments are given the notation *n*, the sub-alignment constrained by position *i* is referred to as *C*_*sub*_, and the entire MSA is referred to as *N*. The number of *n*_*size*_ subsets for *j* that contain the same amino acid composition as C_sub_ is calculated. For each amino acid, the observed composition for the subset is calculated by

$$\left(\begin{array}{c} N_{r,j}\\ n_{r,j} \end{array}\right)=\frac{N_{r,j}!}{n_{r,j}!(N_{r,j}-n_{r,j})!}$$

where *N*_*r*,*j*_ is the number of *r* residues in the entire MSA at position *j*, and *n*_*r*,*j*_ is the number of *r* residues in the subset. The total number of possible subsets that have the same composition as *C*_*sub*_ is the product of the combinations and is defined as Ω_j_^<i>^. The probability that a random subset will have the composition of *C*_sub_ is determined by dividing Ω_j_^<i>^ by the total number of subsets in *N*_total_ according to the following equation:

$$L_{j}^{<i>}=\frac{\prod_{r}(\begin{array}{c} N_{r,j}\\ n_{r,j} \end{array})}{\left(\begin{array}{c} N_{total}\\ n_{total} \end{array}\right)}$$

Another statistic calculated to account for varying sized subsets is determined by the following equation:

$$\Lambda_{j}^{<i>}=\frac{\prod_{r}(\begin{array}{c} N_{r,j}\\ n_{r,j} \end{array})}{\left(\begin{array}{c} N_{r,j}\\ m_{r,j} \end{array}\right)}$$

where *m*_r,j_ is the score of a model sub-alignment. This calculates the probability of random sequences of n size having the same amino acid composition as *C*_*sub*_ compared to the probability of the subset of *n* size containing the most likely sequences.

### Multiple sequence alignments

Sequences were gathered from the non-redundant database using PSI-BLAST with default parameters and a 20,000 max target sequence. Full-length CFTR from Homo sapiens (ID:NP_000483.3) was the query sequence for 50 iterations of PSI-BLAST, which yielded 20,000 sequences. CD-HIT was employed to eliminate sequences with >90% identity to other identified sequence, leaving 8838 sequences [17]. 10 subalignments were generated from Clustal Omega, where NBD2 was isolated and 7306 sequences were left after sequences lacking common motifs were deleted (Walker A/B, Q-loop, C-loop, Walker B, H-loop). The MSA was curated for length, where sequences between 245 and 265 amino acids were retained [43]. The final MSA was generated by utilizing Clustal Omega with auto settings, resulting in 5032 sequences. Columns that did not correspond to the reference sequence were removed and positions containing >35% gaps were not considered in the co-evolution analysis [40,43].

#### Perturbation analyses for S1251T, N1303T, D1270N and S1235R

Sequences containing the desired amino acid mutation at a given position were used to generate sub-alignments for perturbation analyses. The S1251T sub-alignment contained 266 sequences out of the original 5,032 sequences. The N1303T, D1270N and S1235R subsets comprised 87, 23, and 636 sequences respectively.

#### Scrambled MSA for NBD2 Full Alignment, S1251T, N1303T, D1270N and S1235R

Columns of alignments were randomized, producing MSAs with unchanged consensus, but scrambled couplings. Randomization was performed in Microsoft Excel by assigning a random number to each position then ordering the column from highest to lowest value. Multiple randomized MSAs were generated for each sub-alignment and the full MSA of NBD2. The average of the highest scrambled score for each alignment was used as the base-line scrambled score for each respective alignment.

### Blocks In Sequences (BIS) Analysis

The Blocks In Sequences analysis, (BIS), was created by Dibb and Carbone to detect blocks, or fragments of co-evolved residues in protein families with a limited number of sequences (~50) and possess high average pairwise identity, for example vertebrate and viral protein families [44,45]. The N1303T, D1270N, and S1251T subsets were analyzed using a webserver implementation of BIS, the BIS^2^ server [44]. Maximum number of errors, or exceptions for identifying the co-evolution signals is denoted as integer D and BIS iteratively computes co-evolution patterns for all dimensions (d) where d ≤ D. P-values were calculated using a Fischer test comparing perfect co-evolution pattern to the one obtained, for details see [44].

### Prediction of stabilizing mutations for NBD2

The method developed by Sullivan and colleagues for identification of stabilizing mutations was employed [40,43]. This method uses conservation and correlation filters for improved predictive power. The relative entropy (RE) and mutual information (MI) must be calculated for a given MSA for this protocol. RE is determined by the following equation:

$$RE=p(x_{i})\ln\frac{p(x_{i})}{q(x_{i})}$$

where *x* is a position, *p(x*_*i*_*)* is the probability of finding amino acid *x* in column *i*, and *q(x*_*i*_*)* is the frequency of amino acid *x*. The yeast proteome was used as a reference for amino acid frequencies as given by Durani [41]. Positions containing >35% gaps were removed and positions with a high conservation (>95%) were filtered out due to the lack of valuable information for calculating the mutual information. Mutual information, in information theory, is the measure of the dependence of one variable on another and is defined as:

$${MI}_{xy}=\sum_{i}\sum_{j}p(x_{i}{,y}_{j})\ln\frac{p(x_{i},y_{j})}{p(x_{i})p(y_{j})}$$

where *p(x*_*i*_,*y*_*i*_*)* is the joint probability of residues *i* and *j* occurring at positions *x* and *y* respectively, while *p(x*_*i*_*)* is the probability of residue *i* at position *x*. Likewise, *p(y*_*j*_*)* is the probability of amino acid *j* at position *y*. RE was computed in Microsoft Excel, while the algorithms written by A. Fodor generated the MI calculations. Positions with RE values below average were discarded and coupled positions with an MI value in the top 1% of *(n*^*2*^*-n)/2*, where n = 254, were removed due to potential hidden coupling. Visual basic for applications was then used to place coupled positions in a single column with the amino acid with the higher number to the left. Duplicates were removed, while MI order was retained. Microsoft excel functions were utilized to order MI positions per the position’s RE value. If the identified amino acids matched the consensus site in the full MSA (>5,000), then that position was removed from the list.

### MutPred score calculations

Mutations predicted to stabilize NBD2 were further examined using the MutPred2 machine learning algorithm that incorporates molecular and genetic data to score the likelihood that a missense mutation is pathogenetic [46]. The S1251T, N1303T, D1270N and S1235R missense mutations were scored singly, or in combination with individual mutations predicted to stabilize NBD2. Mutations known to disrupt CFTR function (S466T, L475Y, W496V, Y517I, C524A, L526A, D537F, Y563V, A566P, P574A, F575T, E583G, H609T, D529F, S573E, I539T, G550E, R553M, R555K), or mutations that are non-CF causing (F508C, G576A, R668C, V754M, L997F, I1027T, R1162L, S1235R, R31C, R75Q, I148T) were also scored for comparison. The full-length human CFTR protein sequence was used for all analyses and the statistical p-value set to ≤ 0.05 with a pathogenic threshold set to 0.8 corresponding to a 5% false positive rate, or 0.68 corresponding to a 10% false positive rate.

### Cβ-Cβ distances vs. algorithms

Cβ-Cβ distances were calculated for all pairs of amino acids (# of positions^2^/2) from pdb files—MalK-NBD2 (PDB: 3DG7) and NBD2 (1207–1436) of Cryo-EM structure of CFTR (PDB: 5UAK)—using VBA and Excel to calculate the distance based on the formula:

$$d(x,y,z)=\sqrt{{(x}_{2}-{x_{1})}^{2}+{(y}_{2}-{y_{1})}^{2}{+(z}_{2}-{z_{1})}^{2}}$$

For all amino acid pairs, the Cβ-Cβ distance was plotted against the score on a given co-evolution algorithm. This was repeated for each pair identified by ELSC, OMES, McBASC, SCA, MI and RE. Since RE is a one-dimensional algorithm, the individual scores of a pair of amino acids were averaged [42]. All positions that were within eight positions of each other were ignored to prevent biases, and positions containing <65% present un-gapped residues were removed [42].

For comparison of the algorithms, p-values were calculated based on a binomial distribution, according to methods determined by Fodor *et al*. We note that the use of the number 75 in the following equation is arbitrary but justified following Fodor’s reasoning [42].

$$\sum_{n}^{75}(\frac{75!}{n!\;(75-n)!})0.5^{n}\times 0.5^{75-n}$$

Of the highest scoring 75 coupled positions identified by each co-evolution algorithm, we counted the number of pairs (*n*) that are below or equal to the median of all Cβ-Cβ distances for that domain. Therefore, this equation gives the probability that an algorithm that chooses coupled positions at random would identify the same number of coupled positions in the top 50^th^ percentile as the given covariance algorithm. Statistical p-values were calculated for the full MSA, S1251T subset, S1235R subset, and N1303T subset.

### Z-score calculation

Covariance scores for each pair of positions for ELSC, OMES, and McBASC were converted to z-scores using CytoNCA plugin in Cytoscape software [47]. Covariance scores from several scrambled MSAs, where consensus was maintained and covariance was randomized, were averaged and converted to z-scores. Randomized subset MSAs were constructed, where a given number of sequences from each taxon (animal, plant, fungi, protist, bacteria) were randomly taken such that the ratio of sequences from each taxon was maintained from the original MSA or subset. The scores for each pair of residues were averaged for several such randomized MSAs, and z-scores were calculated. Boxplots were generated for the original MSA, the scrambled MSA, and the randomized MSA. These calculations were repeated for each subset (1251, 1235, and the full MSA) and covariance algorithm (ELSC, OMES, McBASC). For subset 1303, due to a lower number of sequences and thus an inconsistent number of positions with sufficient data, the randomized subsets were not averaged, and individual box plots were constructed. For the full MSA, the randomized MSA consisted of 2000 sequences randomly picked from the full MSA that contained the same taxon distribution as the full MSA.

## Results

### Statistical coupling analysis of NBD2 of CFTR

**Fig 1:**
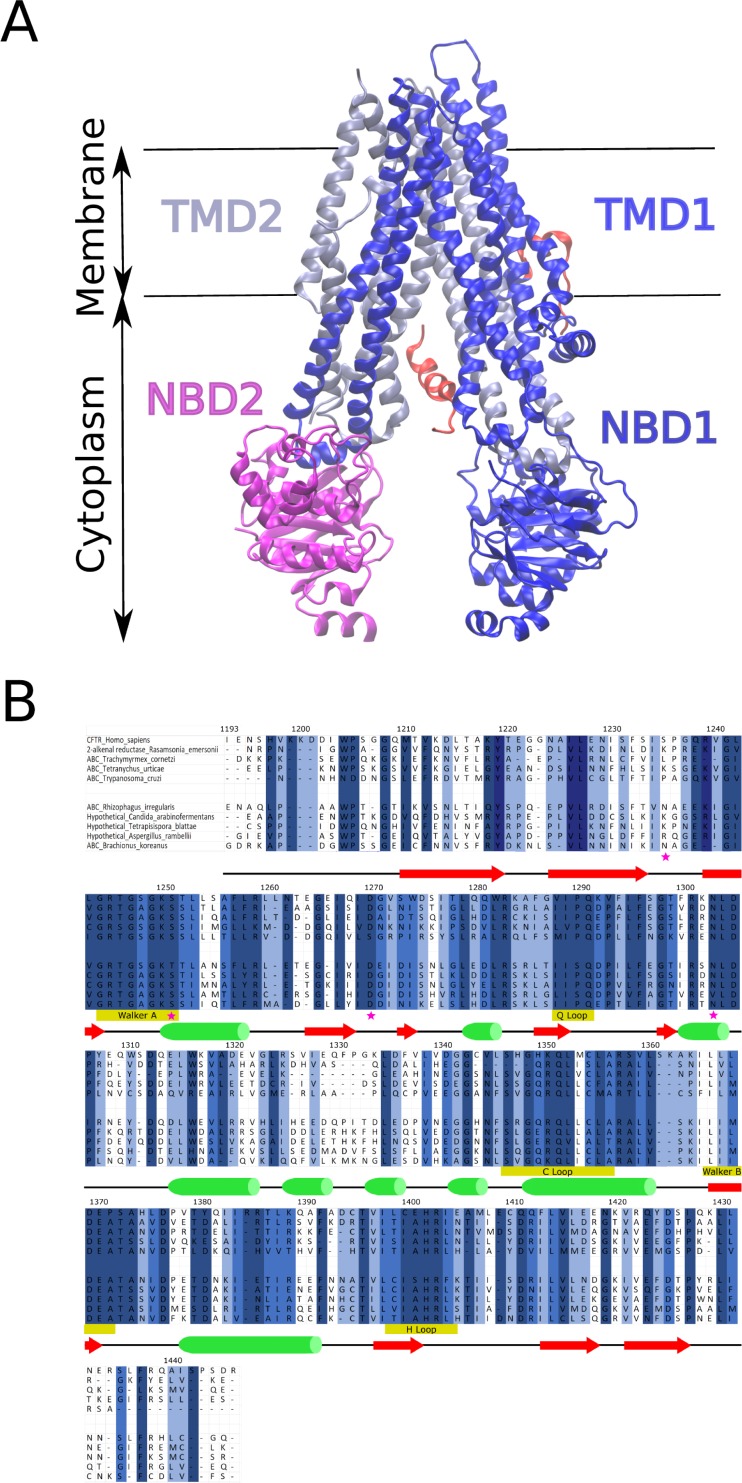
Structure of CFTR protein and multiple sequence alignment of NBD2. (A) The domain structure of the Cystic Fibrosis Transmembrane Conductance Regulator (CFTR) is shown (PDB:5UAK). Transmembrane Domain 1 (TMD1) is ice blue, Transmembrane Domain 2 (TMD2) is dark blue, Nucleotide Binding Domain 1 (NBD1) is dark blue, Nucleotide Binding Domain 2 (NBD2) is magenta, and the Regulatory (R)-domain is red. Important note–the local resolution varies from 2.4 to 6 angstroms (3.9 angstroms overall) and significant portions of the protein were not resolved including residues 1–14, 645–843, 1173–1206 (N-terminus of NBD2), 1437–1480, and the majority of the R-domain [49]. (B) Multiple sequence alignment of conserved regions in NBD2 of CFTR. For visual simplicity, ten sequences out of 11,856 of the alignment are shown. For the whole MSA, residues shaded in dark blue are > 80% conserved. Residues shaded medium blue >60% conserved and residues shaded in light blue are ~ >40% conserved. Secondary structure is depicted below the alignment with green cylinder represent alpha-helix and red arrow represent beta-sheet. Pink star denotes amino acid position identified as mutated in CF patients and investigated by covariance algorithms in this paper. Selected sequences shown are *Homo sapiens*, *Rasamsonia emersonii*, *Trachymyrmex cornetzi*, *Tetranychus urticae*, *Trypanosoma cruzi*, *Rhizophagus irregularis*,*Candida arabinofermentans Tetrapisispora blattae*, *Aspergillus rambellii*, and *Brachionus koreanus*.

**Fig 2:**
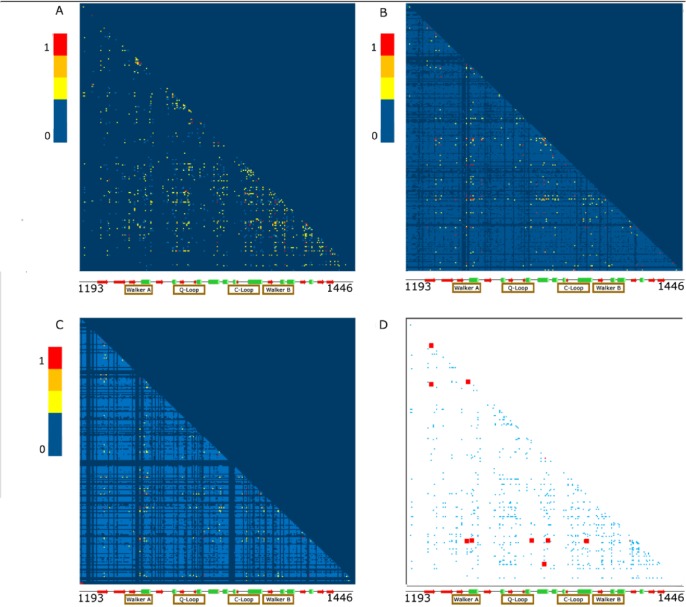
NBD2 matrices for ELSC, OMES, and McBASC analyses. (A) Heat map of the matrix for the McBASC analysis (B) Heat map of the matrix for the ELSC analysis (C) Heat map of the matrix for the OMES analysis (D) Composite heat map depicting high scoring coupled residues (red boxes) identified by all three analyses (ELSC, OMES, McBASC). Cool colors (light blue) represent low coupling scores and warm colors (red) represent high coupling scores. Coupling scores at, or below the average scrambled value were assigned a score of zero and shaded dark blue (See Materials and Methods for details). Matrices are 253 x 253 with the diagonal representing identity. Secondary structure is depicted below the heat maps with same coloring scheme as Fig 1.

Previously, Mendoza and colleagues used co-evolution algorithms to identify residues in CFTR coupled to phenylalanine at position 508 (F508) in NBD1 (Fig 1A)[17]. They found disruption of the network of residues coupled to F508 led to defects in folding and function of CFTR [17]. We performed coevolution analyses to test whether the predicted network of coupled residues in NBD2 of CFTR is disrupted by known CF causing and CF-associated mutations. Homo sapiens CFTR was used as the query sequence for PSI-BLAST of the non-redundant protein database with a 20,000-maximum target parameter, and sub-alignments were performed in Clustal Omega to obtain the NBD2 sequences. The final NBD2 multiple sequence alignment (MSA) contained 5032 sequences and was used in the co-evolution algorithms (Fig 1B). Four algorithms (SCA, OMES, McBASC, ELSC) were used to calculate coupled residue scores for the full MSA of NBD2 (Fig 2 and S1 Fig). Scores were plotted as a 253 x 253 matrix and color coded to produce heat maps of the analyses (Fig 2). To determine a cutoff for evolutionary noise, we scrambled the MSA multiple times and then calculated scores using the four algorithms as described in the Materials and Methods. In general, SCA and McBASC tended to have the least number of positions above the scrambled control. However, ELSC had the highest relative scrambled score. Coupled residue scores falling below the scrambled cutoff were not considered in the subsequent analyses. We graphed each coupled position’s score against its Cβ-Cβ distance to determine the relative power of each algorithm for finding positions that are physically close (S2 Fig)[42]. The Cβ-Cβ distance was calculated from a recent Cyro-EM structure of full-length CFTR (PDB:5UAK), and independently from a crystal structure of a MalK-NBD2 structure (PDB:3DG7), and similar results were obtained (S2 Fig). A statistical p-value was calculated based on a binomial distribution, as discussed in the Materials and Methods, which measures the probability that a coupled position chosen at random would have a Cβ-Cβ distance below the median of all distances. Therefore, smaller probabilities reveal that a given algorithm has a greater probability of identifying positions that are physically close. The p-values for OMES, SCA, and McBASC algorithms were smaller compared to ELSC with better predictive power for McBASC compared to OMES, similar to Fodor’s results (S2 Fig)[42]. Overall, we found the SCA analysis to have one of the highest background noise of the four algorithms, and these results agree with the previous work of Fodor [42]. SCA has a strong bias towards subsets of coupled pairs with one, or more highly variable positions [48]. Therefore, we chose to focus on the OMES, MacBASC, and ELSC analyses for the Full MSA and subsequent perturbation analyses. We also chose to focus our analyses on coupled residue pairs identified by two or more algorithms for two reasons. First, this approach was used by Mendoza and colleagues to identify functionally important coupled positions in NBD1 of CFTR [17]. Second, ELSC, OMES, and McBASC algorithms have varying predictive power for identifying physically close residue pairs and varying strengths for identifying co-evolving residues (S2 Fig) [42,48].

**Fig 3:**
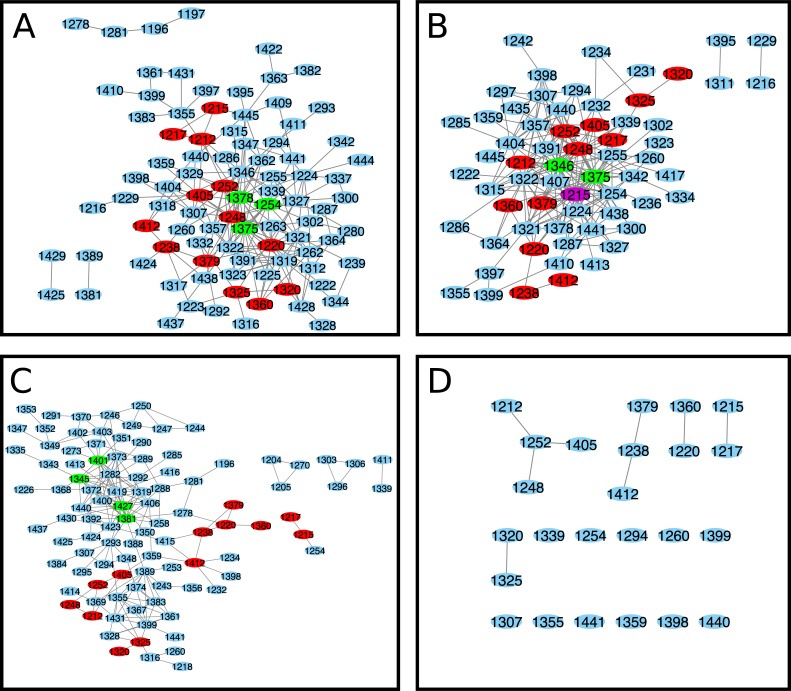
NBD2 Cytoscape interaction maps for matrices. (A) ELSC coupling matrix (B) OMES coupling matrix (C) McBASC coupling matrix (D) Composite of ELSC, OMES, and McBASC analyses. Composite map generated from the individual maps using the top 200 coupled positions common to all (ELSC, OMES, McBASC) using Cytoscape software [50]. Nodes that are central and overlap in all analysis are colored purple. Nodes that overlap in all analysis are colored red, and central nodes are colored green.

To further understand the network relationships uncovered by ELSC, OMES, and McBASC algorithms, we found the 200 highest scoring coupled positions for each algorithm and generated network maps where edges represent covariation signal, and amino acid positions in NBD2 are the nodes (Fig 3)[50]. Nodes that contained the highest number of connecting edges were considered central nodes, or positions to the network and colored green, or purple depending on the number of analyses in agreement (see Fig 3 legend). Residues identified as central positions from ELSC were, L1254, L1375, and L1378 (all numbering and amino acid identities are for the human CFTR protein). Central positions identified by OMES were S1346, and L1375. The McBASC algorithm identified V1345, Q1381, H1401, and I1427 as central positions in the network. Importantly, residue L1375 is a central position in both ELSC and OMES interaction networks. We merged the Cytoscape maps (Fig 3D) to determine the coupled positions in common between the three different analyses. Therefore, the positions shown in Fig 3D are in the top 0.746% of all covariance scores in three independent statistical methods and thus are highly coupled. Two, or more algorithms identified a core set of coupled residues V1212, S1248, and A1405 residues centered around T1252 (Fig 3D). Additional coupled residues in common included T1379, Q1238, Q1412, D1320, and R1325. The identified residues from the composite analysis were located in, or near the Walker A-loop, and H-loop motifs of NBD2.

### Perturbation analyses (S1251T, S1235R, D1270N, N1303T)

Greater than 90% of CF cases are due to deletion of phenylalanine at position 508 (ΔF508), or isoleucine at position 507 (Δ507I) in the NBD1 of CFTR (https://cftr2.org). Both mutations cause non-native residue coupling and aberrant protein dynamics in NBD1 [7]. Much less is known about the rarer NBD2 mutations and the impact of these mutations on NBD2 dynamics. We hypothesized that CF causing mutations in NBD2 would cause non-native residue coupling. This change in the predicted coupling network could be identified through alterations in the central nodes and edges determined by ELSC, OMES, and McBASC analyses (Fig 3D). Furthermore, we predict the pathological severity of the mutation should vary directly with the degree of disruption of the predicted interactions.

To test these hypotheses, we created four perturbations (subsets) to the NBD2 MSA: S1251T, S1235R, D1270N and N1303T. These mutations, or related substitutions were chosen because they account for a significant percentage of non-ΔF508 CF patients and have mild to severe effects on CFTR structure/function. The subset of sequences from the PSI-BLAST containing the desired amino acid mutations at these positions were used to create the new MSAs. For example, the S1235R perturbation analysis was performed by using the 636 sequences—out of the original 5032—of the NBD2 MSA that contained valine instead of isoleucine. The highest 200 scoring coupled positions for each algorithm and perturbation were used to create Cytoscape network maps, and used for comparison with the analyses conducted on the Full MSA [referred to as wildtype from now on, (Fig 3)].

#### S1251T perturbation analysis

The S1251N mutation is prevalent in individuals of European descent and is the most common class III-IV mutation (gating/conductance defect) in the Netherlands with an 1.2% occurrence [32]. The majority of CF patients with this mutation have pancreatic insufficiency. Serine is replaced with asparagine in the conserved Walker A motif of NBD2 leading to reduced ATP binding and hydrolysis. The number of sequences in our full, or wildtype MSA did not contain any sequences with asparagine at position 1251, but there were 266 sequences that contained a threonine at position 1251 instead of serine (S1251T). It is known that the Walker A consensus motif found in ABC transporters, ATP- and GTP-hydrolyzing enzymes possess a conserved serine, or threonine (GXXXXGK(**S/T**)). It is therefore assumed that this conservative substitution of serine with threonine would have minimal impact on ATPase activity. However, this may not be the case and in fact mutation of the serine to threonine in the NBD2 of the ABC transporter p-glycoprotein (S1073T) led to altered ATPase activity depending on the divalent metal complexed with the protein [51]. For example, the S1073T had 20% reduced ATPase activity in presence of Mg^2+^ but a 1.8–2.0-fold increased activity was observed in the presence of Ca^2+^ compared to wildtype [51].

**Fig 4:**
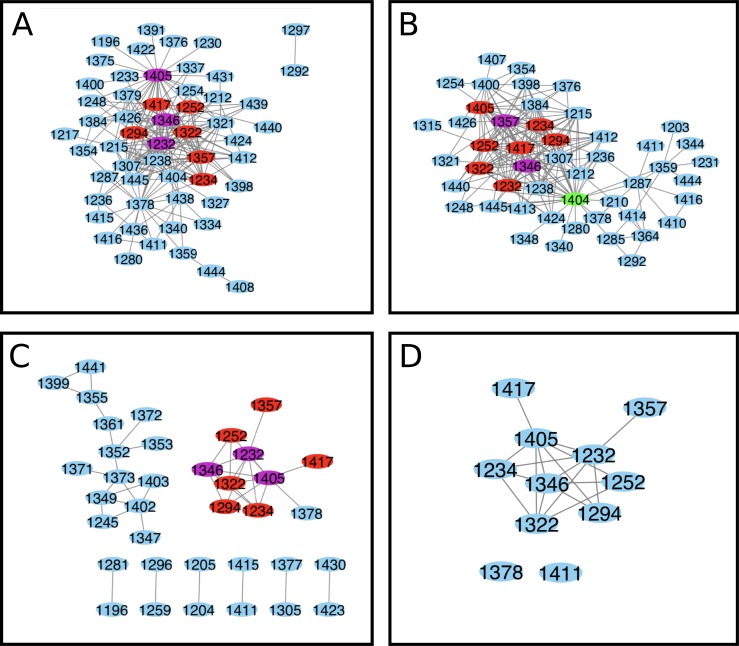
NBD2 Cytoscape interaction map for S1251T perturbation analysis. (A) ELSC (S1251T) coupling matrix (B) OMES (S1251T) coupling matrix (C) McBASC (S1251T) coupling matrix (D) Composite of ELSC, OMES, and (S1251T) analyses. The composite map was generated from the individual maps using the top 200 coupled positions that were common to all analyses (ELSC, OMES, McBASC) using Cytoscape software. Nodes that are central and overlap in all analysis are colored purple. Nodes that overlap in all analysis are colored red, and central nodes are colored green.

We performed co-evolution analysis to investigate the impact of S1251T on the predicted residue coupling in NBD2 of CFTR and hypothesized that this conservative substitution would have a minimal impact on predicted residue coupling. The central positions identified for the S1251T ELSC analysis were F1232, L1346, and E1405 (Fig 4A and S3 Fig). Residues L1346 and R1357 were identified as central for the OMES analysis (Fig 4B). While, residues F1232, L1346, and E1405 were identified as central nodes for the McBASC analysis (Fig 4C). Two, or more algorithms predicted a core set of coupled residues that included F1232, I1234, T1252, F1294, V1322, L1346, and E1405 (Fig 4D). Surprisingly, the only residues in common with the merged wildtype analysis were the predicted coupling between T1252 and E1405. Interestingly, the S1251N and S1251T mutations in CFTR are predicted to be pathogenic according to mutpred2 analysis with scores of 0.816 and 0.719, and both mutations are predicted to disrupt catalytic activity at K1250 with statistically significant p-values of 2.2 x 10^−3^ and 6.6 x 10^−3^, respectively. Importantly, mutation of residue 1250 to alanine (K1250A) is known to abolish ATP hydrolysis because the residue is located in the ATP-binding pocket of NBD2 [52,53].

#### S1235R perturbation analysis

**Fig 5:**
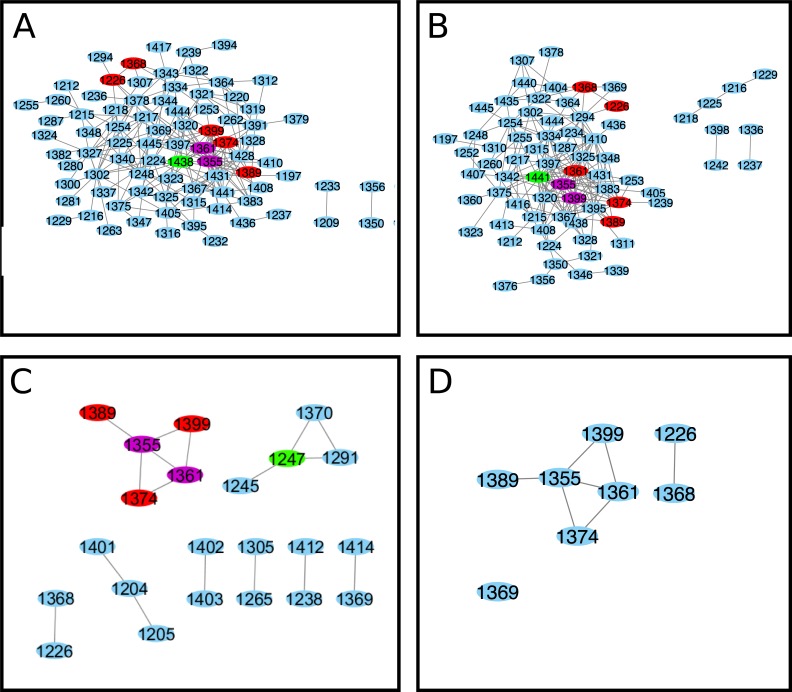
NBD2 cytoscape interaction map for S1235R perturbation analysis. (A) ELSC (S1235R) coupling matrix (B) OMES (S1235R) coupling matrix (C) McBASC (S1235R) coupling matrix (D) Composite of ELSC, OMES, and McBASC (S1235R) analyses. Composite map was generated from the individual maps using the top 200 coupled positions that were common to all analyses (ELSC, OMES, McBASC) using Cytoscape software. Nodes that are central and overlap in all analysis are colored purple. Nodes that overlap in all analysis are colored red, and central nodes are colored green.

The S1235R mutation occurs in approximately 1% of CF patients and is phenotypically manifested by chronic pancreatitis [12]. Patients’ sweat test results are considered borderline for diagnosing CF. We performed co-evolution analysis to investigate the impact of this mutation on NBD2 predicted residue coupling. The central positions for the S1235R perturbation based on the ELSC analysis were L1355, S1361, and R1438 (Fig 5A and S3 Fig). In contrast, the central positions for OMES analysis were L1355, C1399, and I1441 (Fig 5B). Finally, the McBASC analysis identified G1247, L1355, and S1361 residues as being central (Fig 5C). Two, or more analyses identified a core set of coupled residues that included I1226, L1335, S1361, L1368, H1374, Q1389, and C1399 residues.

#### D1270N and N1303T analyses

**Fig 6:**
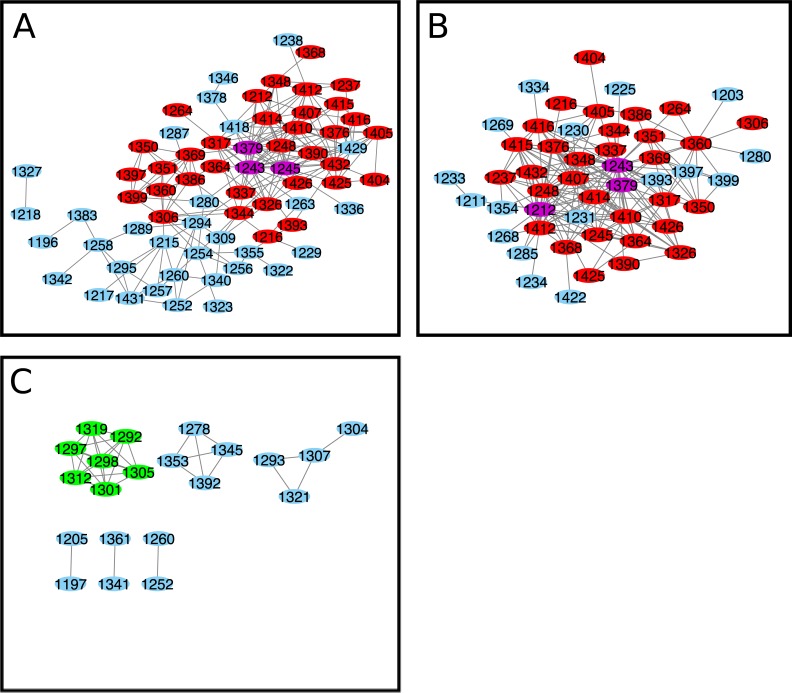
NBD2 cytoscape interaction map for N1303T perturbation analysis. (A) ELSC (N1303T) coupling matrix (B) OMES (N1303T) coupling matrix (C) McBASC (N1303T). Nodes that are central and overlap in all analysis are colored purple. Nodes that overlap in all analysis are colored red, and central nodes are colored green.

The D1270N mutation causes a selective gating defect in CFTR shifting the channel from chloride to bicarbonate preferring [29]. The N1303K mutation is the fourth most prevalent mutation among CF patients worldwide [27]. Unfortunately, there were an inadequate number of K1303 sequences (3) in our MSA to perform the N1303 to K1303 perturbation. However, there were 87 sequences that had T in place of N at position 1303; we used this subset to probe mutation at the 1303 position in NBD2 (N1303T). The ELSC covariation analysis identified L1243, R1245, and T1379 as central positions (Fig 6A and S3 Fig). The OMES analysis identified V1212, L1243, and T1379 as central positions (Fig 6B). The McBASC analysis identified several groups of coupled residues, including K1292, S1297, G1298, R1301, D1305, D1312, and A1319 that contained the most interactions (Fig 6C). Based on our criteria, a composite cytoscape map could not be created because there was no overlap between the three analyses. ELSC, OMES, and McBASC analyses could not be performed for the D1270N MSA because it only contained 23 sequences.

**Fig 7:**
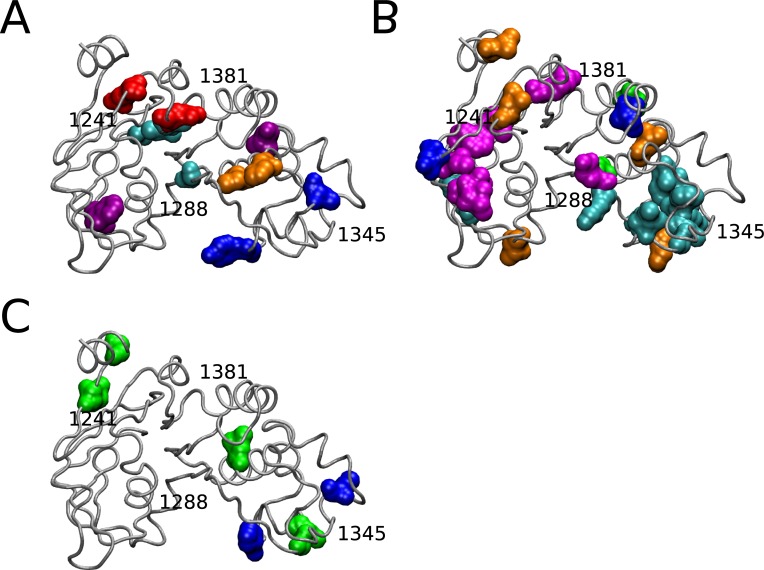
Visualization of BIS results for D1270N, N1303T, and S1251T analyses. (A) Cryo-EM Structure of NBD2 residues 1207–1436 (PDB:5UAK) with BIS predicted co-evolved residues for D1270N with two clusters visualized in D = 0 ((cluster 1 = red; p-value = 0.00073), (cluster 3 = purple; p-value = 0.00075)), and three clusters in D = 1 ((cluster 2 = cyan; p-value = 0.050),(cluster 5 = orange; p-value = 0.0476), (cluster 6 = blue; p-value = 0.00014)) (B) co-evolved residues for N1303T with five clusters visualized in D = 1 ((cluster 1 = cyan; p-value = 0.0179), (cluster 3 = orange; p-value = 0.0167), (cluster 4 = magenta; p-value = 0.0313), (cluster 5 = blue; p-value = 0.0119), (cluster 6 = green; p-value = 0.0116)). (C) co-evolved residues for S1251T with two clusters visualized in D = 1 ((cluster 1 = blue; p-value = 0.00413), (cluster 2 = green; p-value = 5.0612E-08)) Amino acid numbers for reference. Structures visualized using VMD [55] and rendered using Tachyon [56].

The N1303T and D1270N MSAs had reduced number of sequences, 87 and 23 respectively, and these small datasets are not optimal for use with ELSC, OMES, and McBASC analyses. However, Blocks In Sequences (BIS) analysis is specifically optimized to detect coevolution patterns for MSAs with reduced numbers of sequences (around 50 or less) that have low evolutionary divergence that is typically found in vertebrate and viral protein families [39,44,54]. BIS analysis is a combinatorial method that provides a coevolution score for pairs of positions in an MSA and clusters positions with similar coevolution scores. Five clusters of co-evolved residues were identified for D1270N (Fig 7A) and N1303T (Fig 7B). The clusters identified for D1270N were 1401–1412 (red), 1274–1322 (purple), 1307–1345 (blue), 1348–1355 (orange), and 1208-1287-1396-1397 (cyan)(Fig 7A). The clusters identified for N1303T were 1359–1387 (green), 1384–1419 (blue), 1243-1278-1302-1353-1431 (orange), 1215-1217-1292-1295-1298-1299-1300-1301-1305-1312-1315 (cyan), and 1226-1227-1230-1232-1234-1239-1291 (magenta)(Fig 7B). Several of the predicted clusters for D1270N and N1303T span across the α-helical and β-subdomains of NBD2 (Fig 7A and 7B). Residues Q1412, Y1307, and L1355 identified in the D1270N BIS analysis were also identified in the wildtype covariance analyses. Interestingly, five of the seven residues identified as part of a central group in the McBASC analysis for N1303T, also appeared in cluster 1 of the BIS analysis (1292-1297-1298-1305-1312). The only residue in common between the N1303T BIS analysis and wildtype was residue V1359. Interestingly, the D1270N and N1303T BIS analyses predict extensive intra-molecular interactions between the α-helical and β-subdomains of NBD2, and this agrees with the ELSC, OMES, and McBASC analyses for wildtype, and S1251T datasets. However, the analyses do differ in the identity of the individual coupled-nodes, or residues identified in the α-helical and β-subdomains of NBD2.

We attempted to run the BIS analysis for the wildtype and S1235R MSAs, but the clusters identified were not statistically significant (p-value = 1). This is most likely because the algorithm is optimized for MSAs with small number of sequences with relatively high average pairwise identity [39]. However, we were able to run the BIS analysis for the S1251T MSA subset that contained 266 sequences. BIS Clusters identified for S1251T were 1306–1334 (blue), and 1298-1299-1356-1423-1426 (green)(Fig 7C).

#### Z-score distribution for ELSC, OMES, and McBASC analyses

We generated box plots of z-scores to compare the results of the different co-evolution algorithms (ELSC, OMES, McBASC). This technique was used by Pele and colleagues and they chose to focus their analysis on the covarying pairs representing the top 1% of all pairs and thus focus on the tail of their Z-score distribution [48]. Z-scores were generated for scrambled values, randomized sequences, and the original MSAs. We hypothesized that the scrambled values would have, overall, lower z-scores than either the original MSA or the randomized MSA. Additionally, we predicted that if the MSA size does not largely affect the significance of a given covariance score, then the z-score range for randomized subsets would be similar to the full MSA score ranges. If the score ranges are similar, it could possibly indicate a partial independence from specific MSA sequence compositions in terms of the statistical significance of the scores. The randomized subsets appeared to be similar in distribution to the original subset with the exception of McBASC for the full MSA (S7 Fig). Scrambled values tended to be below either the original or the randomized subsets, aside from the McBASC scores. Importantly, the highest Z-scores at the tail-end of the distribution for the scrambled and randomized MSAs were still well below the top values for the original, or experimental MSAs. Similar to Pele, we have chosen our analysis to focus on the top outliers that have Z-scores higher than 4.0. For reference, a Z-score greater than 4.0 corresponds to a p-value < 10^−4^ for a Gaussian Distribution. The Z-score distributions have varying tails but the coupled values we focus our analysis on are well separated from the “noise”.

#### The effect of multiple sequence alignment size on co-evolution analysis

**Fig 8:**
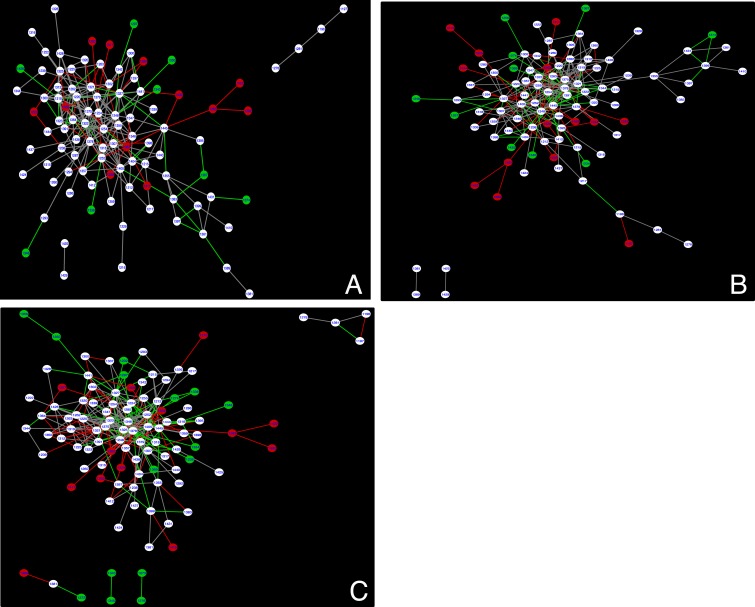
ELSC analysis of Wildtype MSA subsets. Overlay of cytoscape maps for Wildtype (5,032) and (A) 2000, (B) 1000, and (C) 500 sequence MSA subsets, respectively. The original Wildtype MSA is colored red and the subset is colored green with overlapping nodes and edges colored white and grey, respectively. Maps are representative of analyses that were done in triplicate.

The number of sequences vary greatly between the wildtype (5,032) and mutant subset S1235R (636), S1251T (266), N1303T (87), and D1270N (23) MSAs. Co-evolution analysis (ELSC) was performed on randomly selected subsets of the wildtype MSA in order to determine if the MSA size alone could account for the differences observed in the network of predicted couplings for S1235R and S1251T mutant subsets. Subsets chosen for analysis contained the same taxonomic distribution of sequences as the original 5,032 MSA, and three random MSAs were analyzed with sizes ranging from 2000 to 500 sequences (Fig 8 and Table 1).

**Table 1:** Comparison of wildtype MSA to subsets of varying size.

	2000 Sequences	1000 Sequences	500 Sequences
MSA 1	165	148	133
MSA 2	166	153	137
MSA 2	160	153	134
Average	163.67	151.33	134.67
StDev	3.21	2.89	2.08

Number of coupled positions (edges) that overlap with the full MSA (5032 sequnces)

The majority of the coupled residues in the wildtype subset overlap with the original even if the MSA size is reduced four-fold (Fig 8). In total, 8 of 9 analyses have overlap of the core 1212-1252-1248-1405 coupled residues. There is a slight decrease in the overall number of coupled residues as the MSA is reduced from 2000 to 500 sequences (163 versus 134). In comparison, subsets S1251T, S1235R, and N1303T only had 26, 54, and 7 coupled positions that overlap with the full MSA, respectively. There are some differences in the connections (edges) between nodes but the core interactions remain unchanged suggesting the changes seen in the central nodes identified for the S1235R and S1251T mutants compared to wildtype are not simply due to differences in the size of the MSA used for analysis. Furthermore, the highest scoring coupled positions for the wildtype ELSC, OMES, and McBASC analyses also were high-scoring for eigenvector centrality scores (S1 Table). Eigenvalue centrality scores have been used to identify residues at key structural locations in proteins [57]. These results strongly suggest that the highest scoring coupled residues identified by the co-evolution algorithms are predicted to play critical roles in protein function and could be sensitive to mutation.

## Mapping of coupled residues to the NBD2 Structure

**Fig 9:**
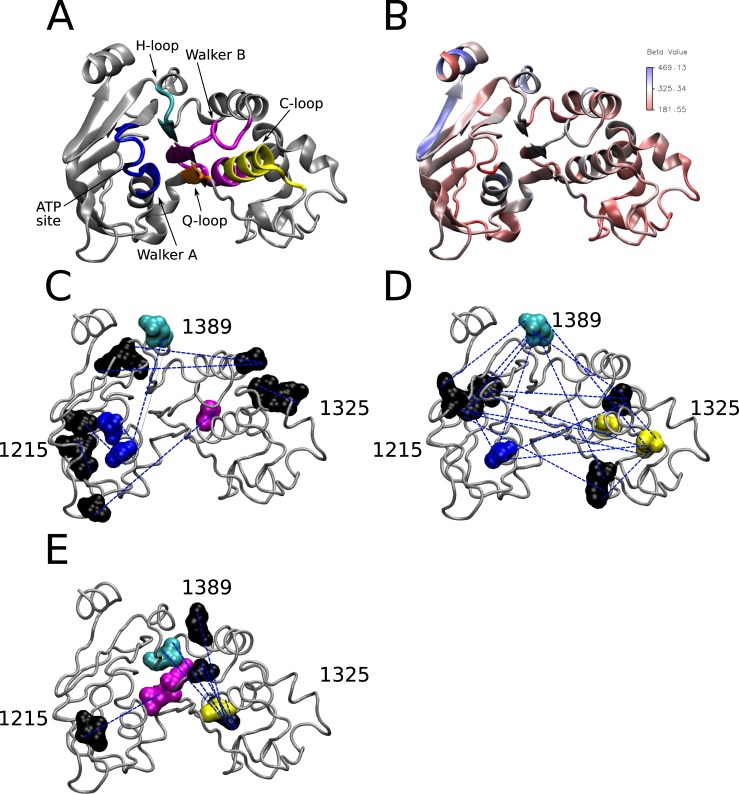
CF mutations alter predicted coupling between α-helical and β-subdomains of NBD2. (A) Cryo-EM Structure of NBD2 residues 1207–1436 (PDB:5UAK) with Walker A (blue), Walker B (magenta), Q-loop (orange), ABC signature C-loop (yellow), and H-loop (cyan) labeled (B) NBD2 structure with residues color coded to reflect corresponding B-Factor values (C) NBD2 wildtype (full MSA) coupled residues from Fig 3D mapped onto NBD2 structure (D) Perturbation S1251T coupled residues from Fig 4D mapped onto NBD2 structure (E) Perturbation S1235R coupled residues from Fig 5D mapped onto NBD2 structure. Coupled residues colored to Walker A, Walker B, Q-loop, H-loop, and C-loop motifs from (A) except for black residues outside aforementioned motifs. Dotted blue lines indicate coupling between residues. Structures visualized using VMD [55] and rendered using Tachyon [56].

We mapped the nodes and edges identified from the wildtype composite network map (Fig 3D) onto the NBD2 structure (corresponding to residues G1207-L1436) of the recently solved cryo-electron microscopy (cryo-EM) structure of CFTR in the ATP-free state to determine if the coupled positions identified were physically or functionally coupled (Fig 9A). Interestingly, the coupled positions reside in or near regions that are predicted to have greater flexibility based on higher B-Factor values (~325 Å^2^) (Fig 9B). Almost all of the highly coupled positions identified in the composite network map for the wildtype (full MSA) analysis were not physically connected based on the 8 Å cutoff proposed by Critical Assessment of Protein (CASP) (Fig 9C and S2 Fig). Coupled residue pairs within bonding distance were 1320–1325 and 1215–1217 with distances of 4.92 Å and 6.59 Å respectively. However, the solved structures for NBD2 only represent one conformation and NBDs are known to undergo a large rigid body rotation upon ATP binding [13,58,59]. Therefore, it is possible that coupled residue pairs identified that are > 8 Å based on these current structures could be brought closer together upon conformational rearrangements. Based on these results, there are groups of residues near the Walker B motif that are predicted to be coupled to residues near the Walker A motif (Fig 9C). There is also predicted coupling between residues near the Walker B motif and residues near the ATP site. Residue A1405 in the H-loop is predicted to have intramolecular interactions with residues in the α-helical and β-subdomains of NBD2. Recently, a Cryo-EM structure of ATP-bound human CFTR was solved (PDB: 6MSM), and we determined if the distances between coupled residues was significantly different between the ATP-free and the ATP-bound conformations [60]. The average RMSD between the backbone of NBD2 from the two CFTR conformations was measured to be 2.393 Å (S8 Fig). We observed distance changes of 2–4 Å between some coupled residues, but residue pairs separated by greater than 8 Å in absence of ATP remained so in presence of ATP (S2 Table).

Next, we mapped the highly coupled positions from the perturbation analyses to determine if these positions are physically interacting and if they were similar to the wildtype. The S1251T perturbation predicted coupling between the Walker A and H-loop motifs (1252–1405) that was also predicted for the wildtype network. Predicted coupling between the α- and β-subdomains was more extensive compared to wildtype (Fig 8D). Notably there were numerous predicted couplings between Walker A (T1252), H-loop (A1405), and C-loop (S1346) motifs compared to wildtype.

The S1235R perturbation contained no central positions in common with wildtype and S1251T, and there was a loss of key coupling interactions between α- and β-subdomains (Fig 8E). There was predicted residue coupling between the C-loop (S1361) and the H-loop (C1399). Predicted coupling between I1226-L1368 was unique to S1235R compared to S1251T, D1270N, N1303T, and wildtype networks. All coupled positions mapped are above the CASP inter-residue distance cutoff suggesting there is no physical but instead a functional, or energetic interaction.

The D1270N BIS analysis identified several clusters of coevolved residues but only residues in cluster five were separated by less than 8 Å (Fig 7A). Interestingly, residues in clusters three and six span the α-helical and β-subdomains of NBD2, that is a recurring theme seen in the wildtype and S1251T analyses. Coevolved clusters were identified in the H-loop (1401–1412) and C-loop (1348–1355). The coevolved clusters are in close proximity but different than the coupled residues predicted by ELSC, OMES, and McBASC for the wildtype, S1251T, and S1235R analyses.

The N1303T BIS analysis identified five clusters of coevolved residues, and the majority of residues in cluster 1 were within 8 Å distance suggesting potential bonding between residues (Fig 7B). Interestingly, co-evolved residues were only predicted in Walker B and none of the other motifs (Walker A, H-loop, C-loop, Q-loop, ATP site), unlike D1270N BIS results. The S1251T BIS analysis identified two clusters, one in the α-helical subdomain and the other that spanned the two subdomains. Both N1303T and S1251T BIS analyses identified coupling between 1298–1299 residues in cluster one and cluster two, respectively. Finally, all the identified clusters had residues that spanned the α-helical and β-subdomains of NBD2 similar to the other analyses, suggesting extensive intramolecular interactions.

Overall, the network of coupled residues is more similar between the wildtype and S1251T analyses compared to the other perturbations, and all the analyses predicted residue interactions between the α-helical and β-subdomains of NBD2. The S1235R is a non-CF causing mutation that leads to elevated levels of pancreatitis in patients [61]. Differences in the residue networks between S1235R and D1270N are unexpected given the close proximity of these two residues on the surface of NBD2. Especially given the fact that both D1270N and S1235R mutations disrupt the bicarbonate conductance ability of CFTR [29]. We would have predicted greater similarity between D1270N and S1235R networks of coupled residues. Finally, the N1303T perturbation had the least overlap with wildtype and this was expected based on the severity of disease that patients with the N1303K mutation exhibit [27].

### Prediction of stabilizing mutations to correct NBD2 network alterations

**Fig 10:**
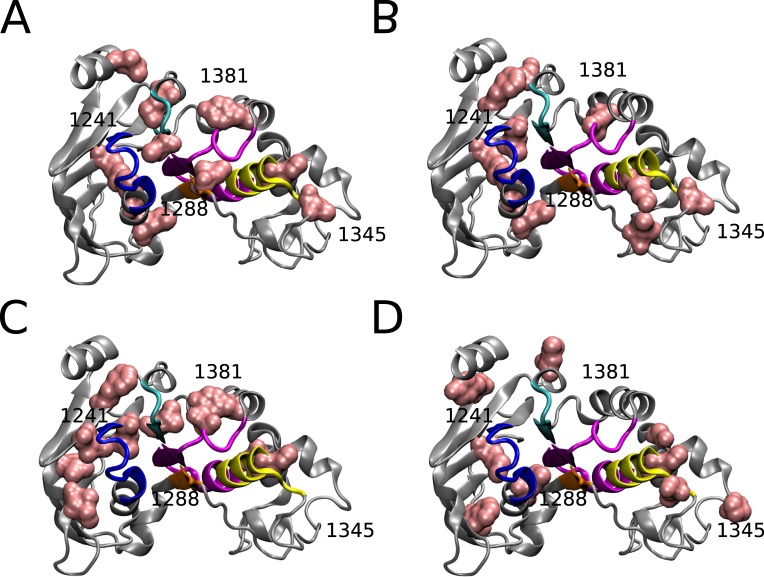
Predicted stabilizing mutations to restore residue coupling in NBD2. Cryo-EM Structure of NBD2 residues 1207–1436 (PDB:5UAK) with predicted stabilizing mutations (see Materials and Methods) colored in pink for (A) S1251T perturbation (B) S1235R perturbation (C) D1270N perturbation and (D) N1303T perturbation. Residue numbers for orientation. Structures visualized using VMD [55] and rendered using Tachyon [56].

We sought to identify stabilizing mutations that could reverse the changes observed in the perturbed coevolution networks. Using Relative Entropy and Mutual Information, we predicted stabilizing mutations for each perturbation (S1251T, S1235R, D1270N, N1303T). Positions with a low MI and high RE value were classified as stabilizing mutations according to Sullivan and colleagues’ method (see Materials and Methods and [40]). We identified 52, 47, 43, and 32 mutations for S1251T, S1235R, D1270N, and N1303T, respectively, that met the criteria for stabilizing mutations. Predicted stabilizing mutations M1210I, F1257L, L1258F, and V1345N were identified for both S1251T and D1270N (Fig 10A and Table 1). The M1210I mutation was also identified for N1303T. Mutations V1212F and L1399T were also identified to stabilize D1270N mutant. The wildtype co-evolution analysis predicted residue V1212 coupling to T1252 and was part of a network involving A1405 and S1248 (Fig 3). Mutation L1254A is predicted to stabilize N1303T mutant and this residue was identified in the wildtype analysis (Fig 3 and Table 2). Not surprisingly, many of the stabilizing residues identified were in, or near the H-Loop, C-Loop, Walker A, and Walker B motifs (Table 2). The N1195P and P1332F mutations are predicted to stabilize the N1303T mutant, but these positions have a higher percentage of gaps in the wildtype MSA compared to the 1303 subset.

**Table 2:** Predicted stabilizing mutations.

1251 Perturbation	1235 Perturbation	1270 Perturbation	1303 Perturbation
**F1257L**	M1210I	L1399T	L1408I
**Y1381D**	F1413V	L1242I	H1350E
**I1289V**	V1345N	V1212F	Y1424F
**H1350Q**	I1295L	Y1381D	N1195P
**I1427P**	L1242I	H1350Q	W1274V
**M1210I**	L1388I	V1318A	G1323H
**M1407I**	K1351R	I1226V	L1346F
**L1258F**	Q1411D	I1416M	M1210I
**C1400I**	L1258F	F1413I	P1332F
**V1345N**	F1257L	S1373T	L1254A

The highest 10 predicted stabilizing mutations for the 1251, 1235, 1270, and 1303 perturbations. For each mutation, the first letter corresponds to the amino acid identity in CFTR *Homo Sapiens*, the sequence number refers to the position in CFTR, and the last letter is the consensus amino acid for the >5,000 sequence MSA.

**Fig 11:**
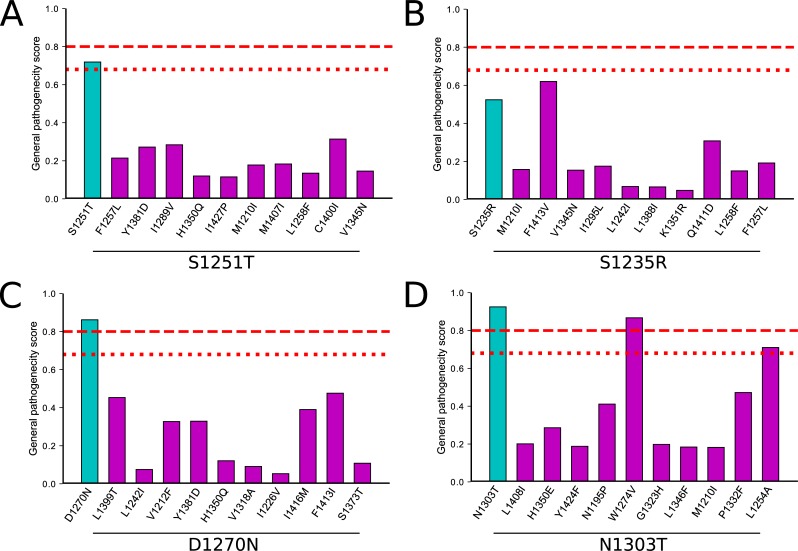
MutPRED analysis of highest ten predicted stabilizing mutations. (A) S1251T perturbation (B) S1235R perturbation (C) D1270N perturbation (D) N1303T perturbation. The general pathogenicity score ranges from zero (benign) to one (pathogenic). A score threshold value of 0.8 for pathogenicity (read dashed line) representing 5% false positive rate, or 0.68 for pathogenicity (read dotted line) representing 10% false positive rate, and P-value threshold of 0.05 was used for analyses.

We used the MutPRED web-based server to predict the pathogenicity of the predicted stabilizing mutations. The MutPRED is a machine learning-based program that incorporates genetic and molecular data to assess the impact a missense mutation will have on the structure/function of a protein [46]. We classified a missense mutation as pathogenetic if the MutPRED score was ≥ 0.68, that yields a false positive rate of 10%, or if the score was ≥ 0.80, that yields a false positive rate of 5%, as recommended by the creators of the program. We measured all missense mutations in the full-length human CFTR protein. First, we measured the impact of the individual S1251T, S1235R, D1270N, and N1303T mutations alone on CFTR. S1235R mutations had score of 0.524, indicating a benign impact on CFTR structure/function. In contrast, the S1251T, D1270N, and N1303T mutations had scores of 0.719, 0.862, and 0.925, respectively, indicating a pathogenic impact. As a comparison, mutations known to disrupt CFTR folding and function [17], and non-CF causing mutations were also measured (www.cftr2.org). The MutPred2 program correctly identified the benign from pathogenic CF mutations with ~82% success rate based on 5% false positive rate (S4 Fig). Next, we measured the predicted top ten stabilizing mutations in each of the four mutant backgrounds (Fig 11). All ten of the stabilizing mutations predicted for the S1251T, S1235R, and D1270N had MutPRED scores similar, or lower than the individual mutations, and none of the scores were ≥ 0.8, suggesting none would negatively impact the function of CFTR (Fig 11). The two mutations predicted to negatively impact CFTR function was mutation W1274V in the N1303T background that scored 0.867 and L1254A that scored 0.710. These results suggest that the majority of predicted stabilizing mutations would not adversely affect CFTR structure/function. Overall, these stabilizing mutations are predicted to restore the network of interactions back to a native-like state and thus improve protein functionality.

## Discussion

### A network of co-evolved residues identified in Walker B, Q-loop, and C-loop regions of NBD2

In this study, we have used five different algorithms (ELSC, OMES, SCA, McBASC, BIS) to identify co-evolved residues in the NBD2 domain of CFTR for both the wildtype and disease-relevant perturbations. As stated earlier, we focused on coupled positions found in two, or more analyses due to the range of predictive power of the algorithms. Another reason to examine several algorithms is because of the differing entropy biases [48]. For example, OMES and ELSC favor pairs of residues with “intermediate” conservation and thus are suited for identifying co-evolving residues [48]. In contrast, McBASC is more suited for identifying residue pairs when the aim is focused on structural information [48].

For the wildtype, coupling was observed between residues in the Walker A, and H-loop. Specifically, the coupling of residues 1252–1405 was observed in the wildtype and S1251T analyses. The results of all analyses suggest predicted intramolecular coupling between the α-helical and β-subdomains. Residues identified in the analyses tended to be in, or around the Walker A, Walker B, C-loop, and H-loop motifs. The extent of predicted interactions varied between the mutant and wildtype analyses. We used the method of Fodor to calculate the probability that choosing coupled positions at random would identify the same number of coupled positions in the top 50^th^ percentile for a given covariance algorithm [42]. The p-value calculated for the OMES and McBASC algorithms were ≤ 0.05 for all MSAs (except 1251, 0.41), and the ELSC datasets had p-values ranging from 5.3 x 10^−2^ (subset 1235) to 1.0 (wildtype). The range of values seen for ELSC is consistent with Fodor’s results [35]. We have confidence based on the calculated p-values that our analyses are statistically robust for OMES and MCBASC, and less robust for ELSC (see Table 3). In addition, the BIS analyses performed for the S1251T, D1270N, and N1303T subsets were also statistically significant with clusters identified with p-values ≤ 0.05 (Fig 7).

**Table 3:** Statistical analysis of ELSC, OMES, McBASC analyses.

MSA	ELSC	OMES	McBASC
Wildtype (full)	1.0	3.2 x 10^−2^	5.3 x 10^−3^
Subset 1251	0.68	0.41	2.6 x 10^−3^
Subset 1235	5.3 x 10^−2^	3.1 x 10^−7^	1.1 x 10^−6^
Subset 1303	0.12	5.4 x 10^−4^	5.4 x 10^−4^

The probability (p-value) above random that the top 75 positions identified by covariance algorithms (ELSC, OMES, and McBASC) are below the median (24.1357 Å) of all Cβ-Cβ distances from the Cryo-Em structure of CFTR (PDB: 5UAK). P-values are calculated using binomial probability (see Materials and Methods).

A previous study by Szollosi and colleagues used a covariance algorithm to identify positions F1296, N1303, and R1358 in NBD2 that were coupled to each other [18]. Our analyses using the ELSC, OMES, and McBASC algorithms did not identify the N1303-F1296-R1358 coupled triad. Position variability is critical for these algorithms to efficiently identify residue coupling. Thus, the failure to identify these residues is not surprising given these positions had high conservation scores in the full MSA. In our hands, performing their version of SCA analysis predicted that F1296, N1303, and R1358 residues are coupled as the frequencies changed upon perturbation, though not to the same magnitude as found previously (S5 Fig)[18]. Our results more closely replicated their dataset if we used an MSA containing either >11,000 sequences, or > 50,000 sequences (S5 and S6 Figs). Using these very large MSAs, asparagine predominated at position 1303, phenylalanine at 1296, and arginine at position 1358 which is in complete agreement with Szollosi and colleagues (S6 Fig). We compared amino acid frequencies for the wildtype and mutants, similar to Szollosi, and our results were in better agreement with their findings using the >50,000 MSA. However, neither >11,000, nor >50,000 sequence MSAs was used in our studies because there were a very large number of truncated, and similar sequences that led to a poor quality MSA that could result in artefacts, including sampling bias [38]. This sampling bias leads to the identification of couplings that arise due to insufficient time for evolutionary divergence instead of functional constraints. We hypothesize that the coupling between N1303-F1296-R1358 residues is weak, and thus not detected in our study as we focused on highly coupled positions above the evolutionary “noise”. Finally, only modest changes in CFTR channel gating were observed upon disruption of the co-evolved triad [18,19].

A study by Gulyás-Kovács used phylogenetic filtering to optimize detection of co-evolving pairs in the NBDs and TMDs of ABC-C transporter family members [19]. CFTR is a member of the ABC-C family and four co-evolving position pairs (1242–1398, 1321–1391, 1389–1409, 1399–1410) in NBD2 were identified in the Gulyás-Kovács study [19]. Our analyses identified 1242–1398, 1321–1391, and 1399–1410 pairs but not the 1389–1409 pair. For example, the 1399–1410 pair ranked 90^th^ in the ELSC and 1242–1398 and 1399–1410 pairs ranked in the top 200 of OMES for the wildtype MSA (Table 4). We also observed several of the Gulyás-Kovács identified coupled pairs in the perturbation analyses, with the N1303T having the lowest ranked pairs compared to the other analyses (Tables 5, 6 and 7). Interestingly, Gulyás-Kovács hypothesized that co-varied residue pairs evolved to regulate protein conformational transitions [19]. Comparison of x-ray crystal structures of NBDs have revealed an ~15° rigid body rotation of the α-helical subdomain towards the β-subdomain upon ATP binding [13, 58, 59]. The Q-loop in NBD2 connects these two domains, has a critical role in ATP binding, and is involved in the conformational orientations observed between the α-helical and β-subdomain found in ABC transporters [14, 62, 63]. The coupling we observed between the H-loop, Walker B, and C-loop motifs may be required for efficient conformational transitions as hypothesized by Gulyás-Kovács and could be needed for the rigid body rotation seen upon ATP binding.

**Table 4:** Full (wildtype) MSA coupled residues in agreement with Gulyás-Kovàcs study[19].

**ELSC**			
***i***	***j***	**Score**	**Rank**
1242	1398	519.0292	250
1321	1391	492.8829	291
1389	1409		
1399	1410	669.7900	90
**OMES**			
***i***	***j***	**Score**	**Rank**
1242	1398	149.6711	27
1321	1391	93.2065	145
1389	1409		
1399	1410	110.4692	78
**McBASC**			
***i***	***j***	**Score**	**Rank**
1242	1398	0.1633	503
1321	1391	0.1497	614
1389	1409		
1399	1410	0.2003	273

The scores and rank for given for coupled positions (*i*,*j*) for each covariance algorithm is shown with the rank representing the coupled positions’ score with respect to all the coupled positions in its covariance algorithm and MSA. For this table, all ranks are out of 26797 coupled positions. The scrambled values for ELSC, OMES and McBASC are 27.2040, 2.2326, and 0.1165 respectively.

**Table 5:** 1251 Subset coupled residues in agreement with Gulyás-Kovàcs study[19].

**ELSC**			
***I***	***j***	**Score**	**Rank**
1242	1398	9.331159	10274
1321	1391	43.40415	1354
1389	1409		
1399	1410	47.06378	1064
**OMES**			
***I***	***j***	**Score**	**Rank**
1242	1398	0.252923	14933
1321	1391		
1389	1409		
1399	1410	3.623822	4184
**McBASC**			
***I***	***j***	**Score**	**Rank**
1242	1398	0.077644	9671
1321	1391	0.427906	97
1389	1409		
1399	1410	0.227894	1501

The scores and rank for given for coupled positions (*i*,*j*) for each covariance algorithm is shown with the rank representing the coupled positions’ score with respect to all the coupled positions in its covariance algorithm and MSA. For this table, all ranks are out of 25,651 coupled positions. The scrambled values for ELSC, OMES and McBASC are 16.5758, 2.3311, and 0.4941 respectively.

**Table 6:** 1235 Subset coupled residues in agreement with Gulyás-Kovàcs study[19].

**ELSC**			
***I***	***j***	**Score**	**Rank**
1242	1398	54.9234	1329
1321	1391	96.7194	161
1389	1409		
1399	1410	84.3776	306
**OMES**			
***I***	***j***	**Score**	**Rank**
1242	1398	18.1643	57
1321	1391	12.9074	264
1389	1409		
1399	1410	24.7473	16
**McBASC**			
***I***	***j***	**Score**	**Rank**
1242	1398	0.2095	360
1321	1391	0.1288	1140
1389	1409		
1399	1410	0.2091	362

The scores and rank for given for coupled positions (*i*,*j*) for each covariance algorithm is shown with the rank representing the coupled positions’ score with respect to all the coupled positions in its covariance algorithm and MSA. For this table, all ranks are out of 31,878 coupled positions. The scrambled values for ELSC, OMES and McBASC are 19.6414, 2.2951, 0.4631 respectively.

**Table 7:** 1303 Subset coupled residues in agreement with Gulyás-Kovàcs study[19].

**ELSC**			
***I***	***j***	**Score**	**Rank**
1242	1398	3.5544	5609
1321	1391	6.9597	2384
1389	1409		
1399	1410	6.9472	2393
**OMES**			
***I***	***j***	**Score**	**Rank**
1242	1398	0.4110	4199
1321	1391	0.5683	554
1389	1409		
1399	1410	1.3869	810
**McBASC**			
***I***	***j***	**Score**	**Rank**
1242	1398	0.1947	5151
1321	1391	0.1574	6077
1389	1409		
1399	1410	0.1446	6392

The scores and rank for given for coupled positions (*i*,*j*) for each covariance algorithm is shown with the rank representing the coupled positions’ score with respect to all the coupled positions in its covariance algorithm and MSA. For this table, all ranks are out of 24,090 coupled positions. The scrambled values for ELSC, OMES and McBASC are 11.0010, 1.8764, 0.9693 respectively.

### CF and CF-related mutations disrupt to varying degrees the predicted NBD2 co-evolution network of residues

We have investigated the changes that occur in the NBD2 co-evolved residue network upon the introduction of CF and CF-associated mutations. There were varying degrees of network alteration from moderate to severe depending on the mutation. The S1251T, D1270N, and S1235R networks were different from the wildtype (Figs 7 and 8). These perturbations had more interactions between H-loop residues, and residues in the α-helical subdomain near the C-loop. In contrast, the wildtype analysis identified interactions between H-loop and Walker A (Fig 8A). The N1303T mutation had a co-evolved cluster centered around residue 1298 that was not seen in the other analyses (Fig 7B) and a pattern of co-evolved residues very different from wildtype. These differences are not unexpected given the N1303K mutant CFTR protein expression level is ~10% of the wildtype and ~95% of patients with this mutation have pancreatic insufficiency, highlighting the importance of this residue for CFTR function [27]. The S1251T network had predicted interactions between the Walker A and H-loop similar to wildtype but additional interactions between the Walker A and C-loop were predicted (Fig 9). The D1270N perturbation had co-evolved clusters of residues in the Q-loop and H-loop, and these motifs are important for efficient ATP hydrolysis and channel closing [64, 65]. Interestingly, patients with the S1235R, or D1270N mutations have pancreatitis but not CF. Of note, S1235R and D1270N mutations have recently been re-classified as mutations that cause a selective bicarbonate defect in CFTR (*CFTR*^*BD*^) and disrupt function of organs that utilize CFTR bicarbonate conductance, such as the pancreas, nasal sinus, and vas deferens [29]. Based on our hypothesis, we would have predicted that the network of interactions should be similar for these two mutations, but surprisingly they are not. Our results suggest there is flexibility in residue interactions such that communication between the α-helical, and β-subdomains of NBD2 are retained. Finally, the differences between the S1235R and D1270N networks suggest the underlying molecular defect for these *CFTR*^*BD*^ variants are different despite the fact that the two residues are near each other on the surface of NBD2.

### Comparison between NBD1 and NBD2 predicted co-evolution network of residues

Recently, Mendoza and colleagues performed statistical coupling analysis and identified 45 residues coupled to F508 in NBD1. They mutated sixteen of the residues coupled to F508 and performed *in vitro* NBD1 structural complementation assays to assess folding/stability. Two of the 508-coupled mutations improved NBD1 folding/stability while the other fourteen 508-coupled mutations impaired it [17]. Six of the coupled residues are in the α-helical domain and ten of sixteen sites are surface exposed. Two of these surface exposed sites (F490 and W496) are predicted to interact with Intracytoplasmic loop 4 (ICL4) of the full-length CFTR. We also observed several coupled positions in NBD2 that lie in the α-helical domain (V1345, S1348, T1381) and are surface exposed based on the CFTR structural models (3GD7 & 5UAK).

Proctor and colleagues identified cross-domain coupling in NBD1 of CFTR using discrete molecular dynamic simulations [7]. Specifically, they observed that deletion of F508, or I507 residues led to increased fluctuations between the regulatory insertion (RI, 404–435), structurally diverse region (SDR, 532–552), F508-loop (507–514), and ATP-binding subdomain (570–600), suggesting dynamic coupling between these affected regions of NBD1 [7]. Similar to Proctor *et al*., we also observed coupling between the ATP-binding region, in the α-helical subdomain and the β-subdomain. Mutation of S942 to proline (S492P) partially rescued the folding defect of ΔF508-NBD1 and ΔI507-NBD1 when a second site suppressor mutation (I539T) was also present in CFTR [7,8]. The S492P mutation appeared to “stiffen” the RI-SDR interactions and thus partially reverse the heightened fluctuations observed upon deletion of I507 residue in NBD1 [7]. Interestingly, CFTR from other species such as chicken contain a proline at the position equivalent to S492 in human CFTR, and the chicken CFTR is more stable compared to human CFTR [8]. Increasing substitution of prolines in the SDR region of NBD1 can enhance stability in ΔF508-NBD1 and restore NBD1-ICL4 interaction [8]. We identified residues in our analyses of NBD2 (wildtype = 1294, N1303T = 1292, 1295, 1298, 1299), and these residues are in close proximity to the P1290 residue that is the equivalent to S492 in NBD1. Importantly, our coupling analysis suggest the allosteric modulation observed for NBD1 could also occur for NBD2. It is intriguing to speculate the stabilizing mutations predicted for the NBD2 mutants (Fig 9) could aid in restoring native-like dynamics through a similar “stiffening” mechanism as seen for NBD1 [8].

### Implications of predicted disruption of co-evolution network on CFTR function

It is not known the effect of mutating serine to threonine at position 1251 in CFTR but mutation of the serine to alanine (S1251A) is known to disrupt CFTR maturation leading to ER retention and degradation in the cell [66]. The MUTPRED2 algorithm predicted that S1251T mutation in CFTR is pathogenic and an analogous mutation in the ABC transporter p-glycoprotein alters ATPase activity [67]. The Walker B motif contains a conserved aspartate (D1370) in NBD2 that is critical for coordination of the Mg^2+^ ion and a conserved glutamate (E1371) that acts as the general base during ATP hydrolysis [68,69]. The α- and β-subdomains are linked by the Q-loop that contains a conserved glutamine (Q1291) that is a phosphate sensor and has a key role in induced fit and the 15° rotation of the subdomains two each other during ATP binding [58]. The histidine at position 1402 (H1402) holds these catalytic residues in the right orientation and this residue has been called the “linchpin” or “switch histidine” [70]. The Wildtype and S1251T have predicted coupling between residues in the Walker A and the H-loop but no predicted coupling with the Q-loop (Fig 9C and 9D). There is no predicted coupling involving the Q-loop in S1235R as well (Fig 9E). This is consistent with the Q-loop needing to be flexible to accommodate the above-mentioned rotation. However, there is predicted coupling between the Q1291 residue and the β-subdomain near the Walker A for N1303T (Fig 7B). This predicted coupling could potentially hamper the induced fit rotation thus leading to the observed severe gating defect, and aberrant response to ATP analogs for this mutant [71]. Interestingly, The BIS analysis for S1251T predicts coupling between the two subdomains but not between the Walker A and the Q-loop (Fig 7C). The D1270N mutation that is not CF-causing but alters bicarbonate preference of CFTR and can lead to pancreatitis is located on the surface of NBD2. There is predicted coupling between the Q-loop and the β-subdomain but this coupling is minimal compared to N1303T. This result suggests that extensive coupling between the Q-loop and β-subdomain could alter the dynamics of NBD2 thus leading to defects in CFTR gating. Overall, there appears to be multiple residues that can participate in coupling between the two subdomains without severely affecting CFTR function. In contrast, altered coupling between the Q-loop and β-subdomain is predicted to be detrimental.

### Predicted stabilization of NBD2 co-evolution network

We performed calculations using the method of Sullivan to predict mutations that would stabilize the predicted co-evolution network of NBD2 [41]. This same approach was used successfully, nine out of ten times, to identify stabilizing mutations for the triosephosphate isomerase protein which was significantly better than the previous 50% success rate based on conservation alone [43]. The majority of the top ten stabilizing mutations we identified in NBD2 were located in the β-subdomain in and around the Walker A motif (Fig 10). The remaining mutations were near the C-loop in the α-helical subdomain. Importantly, all the predicted stabilization mutations except two were predicted to be benign according to MUTPRED2 program (Fig 11).

Many of the predicted stabilizing mutations mapped to the C-loop (Fig 10). These results are in agreement with a recent study that identified stabilizing mutations in NBD2 using a back-to-consensus scoring methodology [20]. Vernon and colleagues identified S1255L, K1292D, S1359A, and K1334G as stabilizing mutations. We identified K1292D as stabilizing but not top-ten scoring for subsets 1235, 1270. Mutation S1359A was identified as stabilizing in our 1234, 1235 perturbation analyses, and K1334G was identified as stabilizing in our 1270 analysis, but none scored in the top-ten highest scoring. Importantly, the predicted stabilizing mutations are predicted to be benign based on MUTPRED2 analysis. These predictions provide a starting point to further probe the allosteric dynamics of NBD2. Of course, prediction of stabilizing mutations needs to be confirmed by biochemical experiments to substantiate these computational findings.

## Conclusion

In summary, we have identified a co-evolution network of residues in NBD2 of CFTR consisting of interactions between the Walker A, H-loop, and residues in close proximity to Walker B and C-loop motifs. The S1251T mutant appeared to have more predicted residue interactions in the α-helical domain compared to wildtype, and between the Walker A and C-loop. These predicted intramolecular interactions between α-helical and β-subdomains is similar to that observed from discrete molecular dynamic simulations of NBD1 of CFTR [7]. The greatest alteration in the residue network compared to the wildtype occurred upon mutation of residue 1303, the substitution of which—from asparagine to lysine—causes CF. Interestingly, the co-evolution networks for the S1235R and D1270N perturbations were dissimilar from one another despite their similar pathological effects, suggesting that the underlying molecular defect for these two *CFTR*^*BD*^ variants are different despite the residues’ close structural proximity. Finally, we have identified potential stabilizing mutations that could restore the predicted disruption in co-evolution network caused by the S1251T, S1235R, D1270N, and N1303T mutations. These results reinforce the notion that intramolecular interactions between the α-helical and β-subdomain are needed to preserve NBD2 folding/function. In contrast, inappropriate interactions between β-subdomain and the Q-loop could be detrimental to NBD2 dynamics and CFTR function. To the best of our knowledge, this is the first in-depth investigation of the impact common CF-causing and CF-associated mutations have on the predicted NBD2 co-evolved residue network in CFTR. Future work will focus on experimentally validating the predictions obtained from these *in silico* studies.

## Supporting information

S1 Fig NBD2 coupling matrices for SCA. Heat maps represent coupled positions identified by the Statistical Coupling Analysis for perturbations (A) Iso^1234^, (B) Ser^1235^, (C) Asn^1303^, and (D) for the wildtype (Full MSA). High scores are represented by warm colors (red), and cool colors depict low scores. Coupled positions at or below the scrambled score for that MSA were colored dark blue.
(PNG)

S2 Fig Comparison of ELSC, OMES McBASC, SCA, MI and RE in identifying physically interacting residues. Distances in angstroms between Cβ-Cβ atoms from the NBD2 domain (1207–1436) of the 5UAK crystal structure are graphed against the covariance scores from (A) ELSC, (B) OMES, (C) McBASC, (D) RE, (E) SCA, and (F) MI. Vertical lines mark the top 75 coupled positions, while the horizontal lines at 24.14 Å and 8 Å represents the median of all Cβ-Cβ distances and the CASP cutoff for residue interactions respectively. P-values are calculated based on a binomial probability (see Methods and Materials), where lower p-values indicated higher algorithm strength. For comparison of crystal structures, p-values were calculated for both 5UAK and 3GD7 and a Homology model of NBD2 (G).
(PNG)

S3 Fig Perturbation analyses for S1251T, S1235R, and N1303T residues of NBD2. Heat maps depicting high scoring coupled residues for perturbation analyses S1251T (A-D), S1235R (E-H), and N1303T (I-L). Algorithms ELSC (A, E, I), OMES (B, F, J), and McBASC (C, G, K) were performed for the perturbation analyses. Composite heat maps (D, H, L) depicting high scoring coupled residues (red boxes) identified by all three analyses (ELSC, OMES, McBASC). Cool colors (light blue) representing low coupling scores and warm colors (red) representing high coupling scores. Coupling scores at or below the average scrambled value were assigned a score of zero and shaded dark blue (See Materials and Methods for details).
(PNG)

S4 Fig Protein pathogenicity and stability predictions for selected CFTR mutations. MutPRED pathogenicity predictions for mutations identified by Mendoza *et al*. [17] to either increase, or decrease the folding yield of NBD1 (magenta bars). For comparison, common non-CF causing mutations were analyzed as a control (cyan bars). The general pathogenicity score ranges from zero (benign) to one (pathogenic). A score threshold value of 0.8 for pathogenicity (red dotted line) and P-value threshold of 0.05 was used for analyses.
(EPS)

S5 Fig Amino acid frequency distributions for MSA with >11,000 sequences. Amino acid frequency distributions at position 1296 (site 1 –Szollosi [15]), 1303 (site 2 –Szollosi [15]), and 1358 (site 3 –Szollosi [15]) in full (wildtype) MSA. Amino acid frequency distributions at position 1303 in 1296 (1296I, 1296Y, 1296L) and 1358 (1296I, 1296Y, 1296L) perturbations.
(DOCX)

S6 Fig Amino acid frequency distributions for MSA with >50,000 sequences. Amino acid frequency distributions at position 1296 (site 1 –Szollosi [15]) and 1303 (site 2 –Szollosi [15]) in full (wildtype) MSA. Amino acid frequency distributions at position 1296 in 1303 perturbations (1303Q, 1358A). Amino acid frequency distributions at position 1303 in 1296S perturbation and 1358 in 1303Q perturbation.
(DOCX)

S7 Fig Boxplots of Z-scores for ELSC, OMES, and McBASC analyses. (DOCX)

S8 Fig **Alignment of NBD2 from ATP-free (PDB:5UAK) and ATP-bound (PDB:6MSM) conformations of human CFTR**, ATP-free NBD2 (cyan) aligned with ATP-bound NBD2 (magenta). Alignment performed and visualized in VMD [55] and rendered using Tachyon [56].
(EPS)

S1 File Raw covariance scores for wildtype (full) analyses. (XLSX)

S2 File Raw covariance scores for I1234V perturbation analyses. (XLSX)

S3 File Raw covariance scores for S1235R perturbation analyses. (XLSX)

S4 File Raw covariance scores for N1303T perturbation analyses. (XLSX)

S5 File Raw covariance scores for randomized controls. (XLSX)

S6 File Multiple sequence alignment used for analyses. (XLSX)

S1 Table Eigenvalue centrality scores for full MSA (Wildtype). (XLSX)

S2 Table Select coupled residue distances for wildtype, I1234V, and S1235R for ATP-free and ATP-bound NBD2 structures. (XLSX)
