# Correction: Disease-relevant mutations alter amino acid co-evolution networks in the second nucleotide binding domain of CFTR

**DOI:** 10.1371/journal.pone.0229986

**Published:** 2020-02-27

**Authors:** Gabrianne Ivey, Robert T. Youker

There is an error in the caption for Fig 1. The correct number of sequences should be 5,032 as listed in the manuscript. Please see the complete, correct Fig 1 legend here.

**Fig 1:**
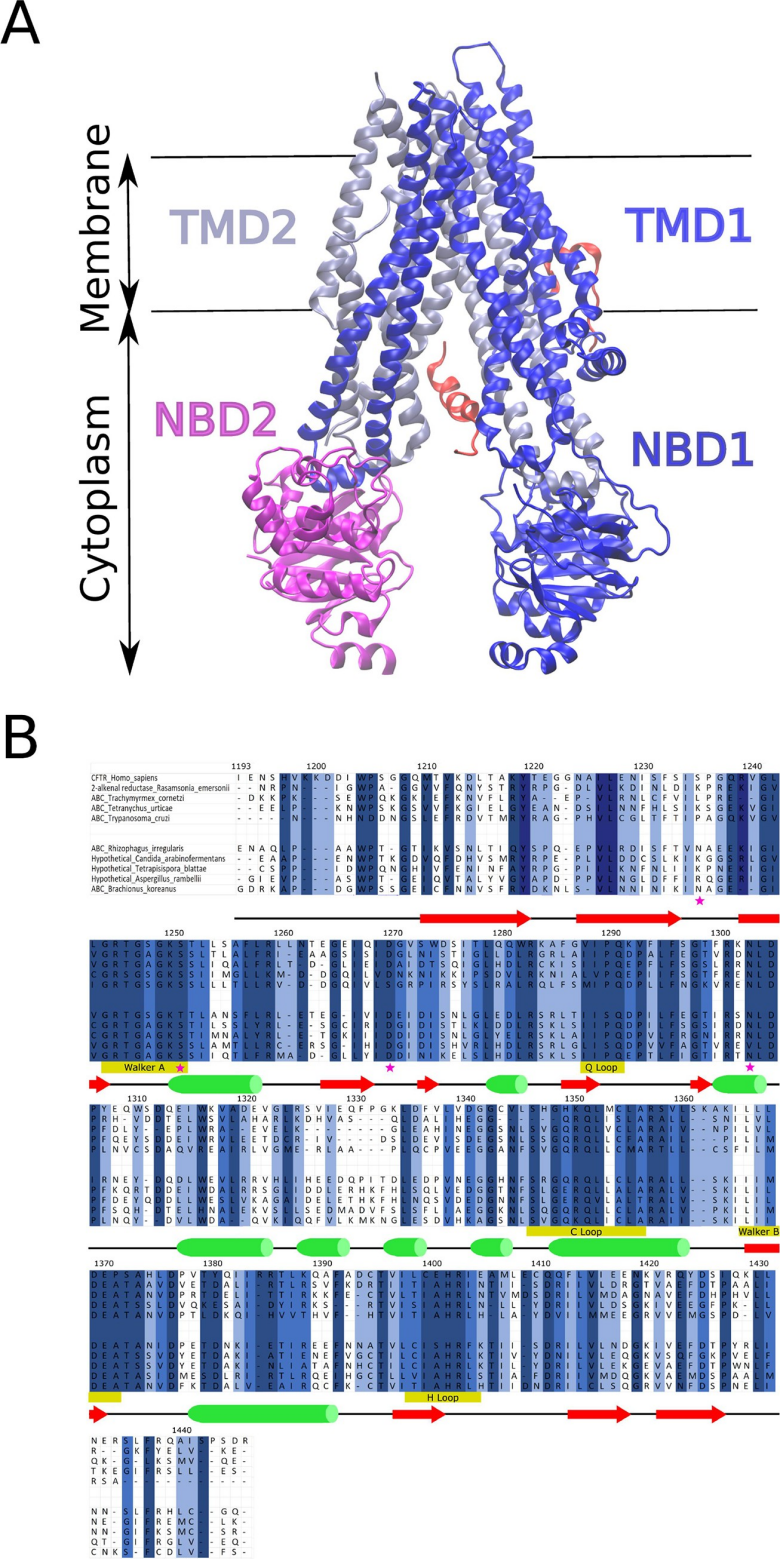
Structure of CFTR protein and multiple sequence alignment of NBD2. (A) The domain structure of the Cystic Fibrosis Transmembrane Conductance Regulator (CFTR) is shown (PDB:5UAK). Transmembrane Domain 1 (TMD1) is ice blue, Transmembrane Domain 2 (TMD2) is dark blue, Nucleotide Binding Domain 1 (NBD1) is dark blue, Nucleotide Binding Domain 2 (NBD2) is magenta, and the Regulatory (R)-domain is red. Important note–the local resolution varies from 2.4 to 6 angstroms (3.9 angstroms overall) and significant portions of the protein were not resolved including residues 1–14, 645–843, 1173–1206 (N-terminus of NBD2), 1437–1480, and the majority of the R-domain [49]. (B) Multiple sequence alignment of conserved regions in NBD2 of CFTR. For visual simplicity, ten sequences out of 5,032 of the alignment are shown. For the whole MSA, residues shaded in dark blue are > 80% conserved. Residues shaded medium blue >60% conserved and residues shaded in light blue are ~ >40% conserved. Secondary structure is depicted below the alignment with green cylinder represent alpha-helix and red arrow represent beta-sheet. Pink star denotes amino acid position identified as mutated in CF patients and investigated by covariance algorithms in this paper. Selected sequences shown are *Homo sapiens*, *Rasamsonia emersonii*, *Trachymyrmex cornetzi*, *Tetranychus urticae*, *Trypanosoma cruzi*, *Rhizophagus irregularis*, *Candida arabinofermentans*, *Tetrapisispora blattae*, *Aspergillus rambellii*, and *Brachionus koreanus*.

There is an error in the caption for S1 Fig. Panel A should be labeled “Ser^1251^”. Please see the complete, correct S1 Fig caption below.

There are a number of errors in the S2 File caption. Please see the complete, correct S2 File caption below.

There are a number of errors in the S2 Table caption. Please see the complete, correct, S2 Table caption below.

## Supporting information

S1 Fig NBD2 coupling matrices for SCA. Heat maps represent coupled positions identified by the Statistical Coupling Analysis for perturbations (A) Ser^1251^, (B) Ser^1235^, (C) Asn^1303^, and (D) for the wildtype (Full MSA). High scores are represented by warm colors (red), and cool colors depict low scores. Coupled positions at or below the scrambled score for that MSA were colored dark blue.
(PNG)

S2 File Raw covariance scores for S1251T perturbation analyses. (XLSX)

S2 Table Select coupled residue distances for wildtype, S1251T, and S1235R for ATP-free and ATP-bound NBD2 structures. (XLSX)
