# Supplementary figures and images for: Disease-relevant mutations alter amino acid co-evolution networks in the second nucleotide binding domain of CFTR
Source: PLoS One. 2020 Jan 24;15(1):e0227668. doi: 10.1371/journal.pone.0227668 (PMC6980524; doi:10.1371/journal.pone.0227668)

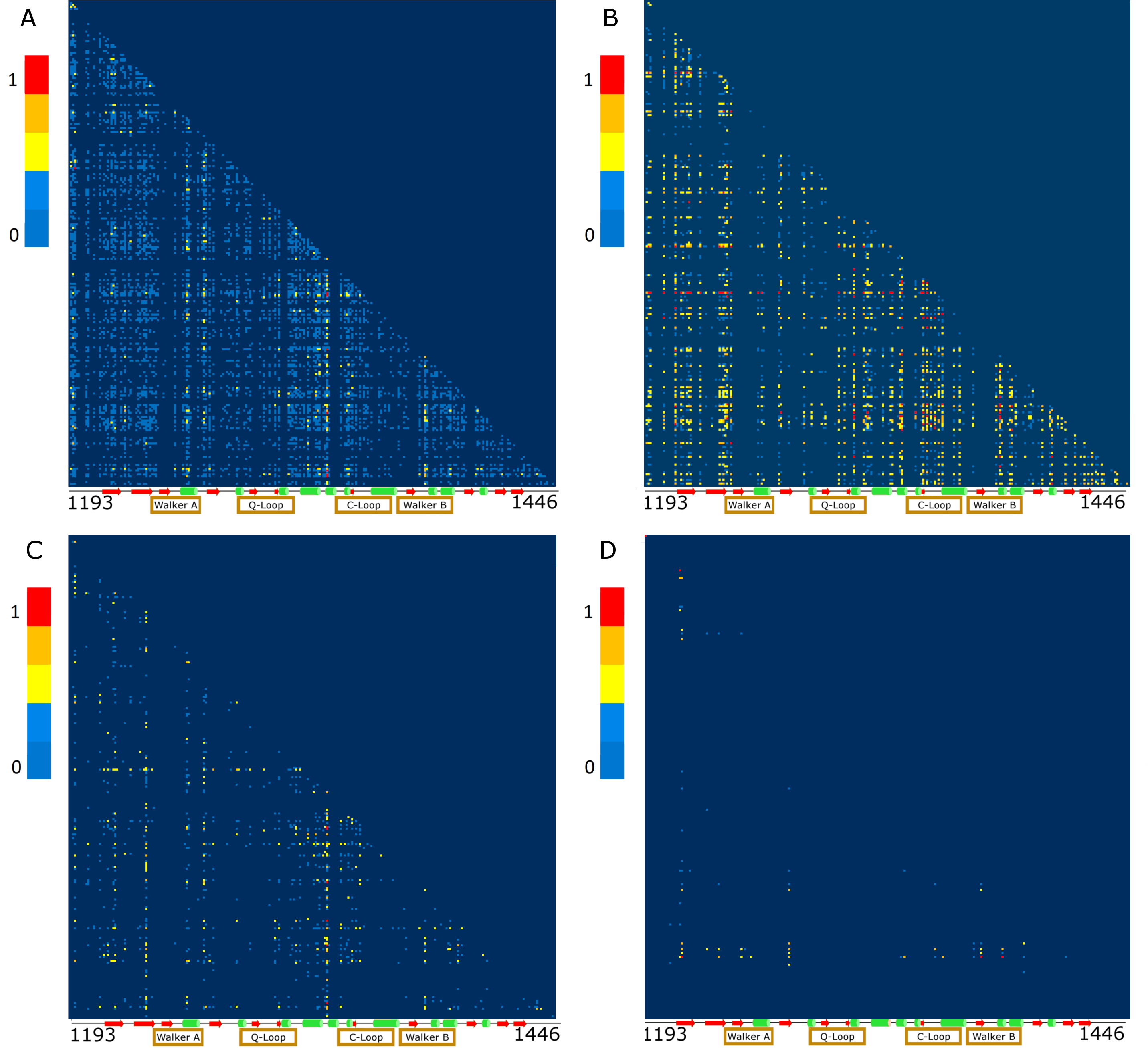

Supplement: S1 Fig — Heat maps represent coupled positions identified by the Statistical Coupling Analysis for perturbations (A) Iso1234, (B) Ser1235, (C) Asn1303, and (D) for the wildtype (Full MSA). High scores are represented by warm colors (red), and cool colors depict low scores. Coupled positions at or below the scrambled score for that MSA were colored dark blue. (PNG) [file pone.0227668.s001.png]

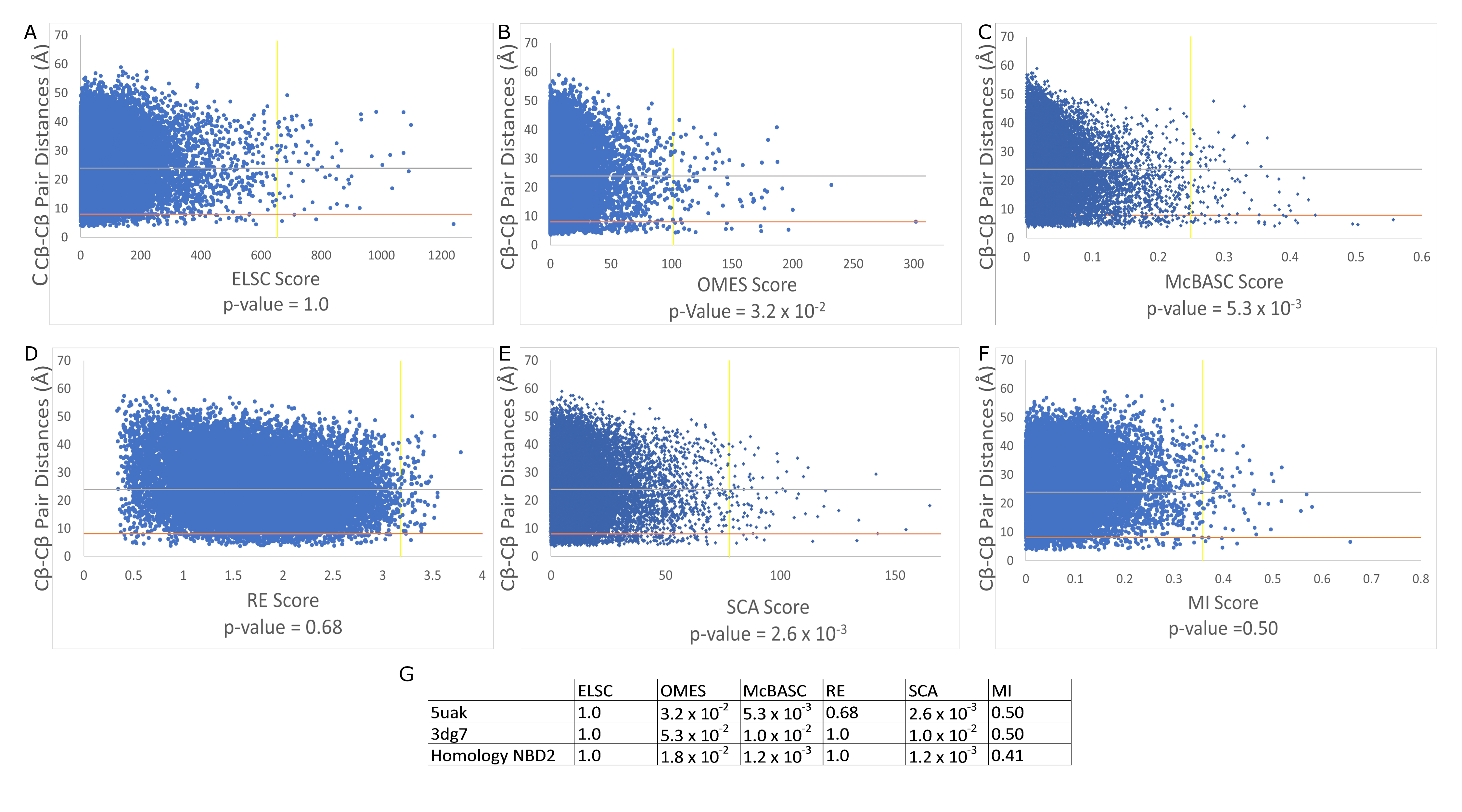

Supplement: S2 Fig — Distances in angstroms between Cβ-Cβ atoms from the NBD2 domain (1207–1436) of the 5UAK crystal structure are graphed against the covariance scores from (A) ELSC, (B) OMES, (C) McBASC, (D) RE, (E) SCA, and (F) MI. Vertical lines mark the top 75 coupled positions, while the horizontal lines at 24.14 Å and 8 Å represents the median of all Cβ-Cβ distances and the CASP cutoff for residue interactions respectively. P-values are calculated based on a binomial probability (see Methods and Materials), where lower p-values indicated higher algorithm strength. For comparison of crystal structures, p-values were calculated for both 5UAK and 3GD7 and a Homology model of NBD2 (G). (PNG) [file pone.0227668.s002.png]

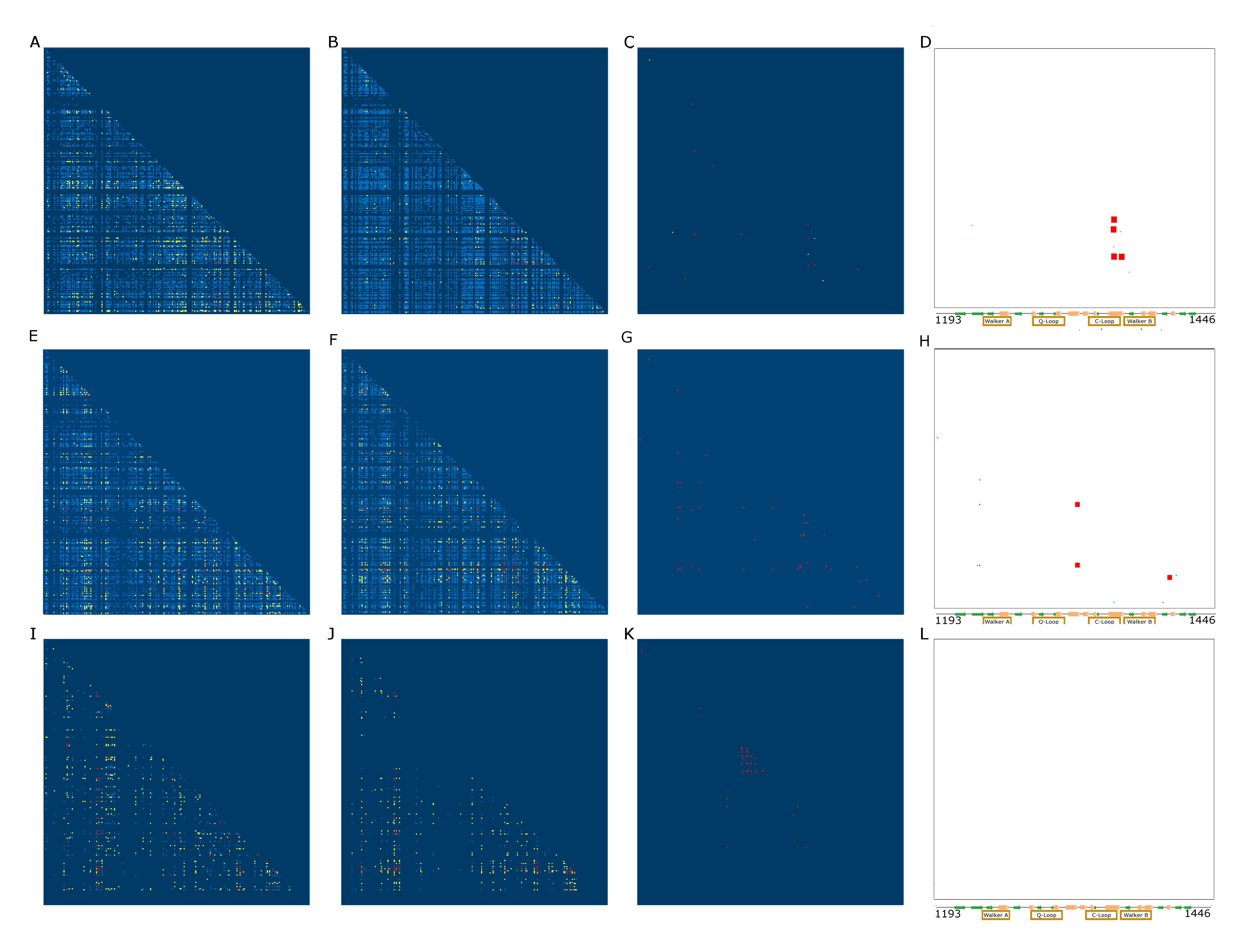

Supplement: S3 Fig — Heat maps depicting high scoring coupled residues for perturbation analyses S1251T (A-D), S1235R (E-H), and N1303T (I-L). Algorithms ELSC (A, E, I), OMES (B, F, J), and McBASC (C, G, K) were performed for the perturbation analyses. Composite heat maps (D, H, L) depicting high scoring coupled residues (red boxes) identified by all three analyses (ELSC, OMES, McBASC). Cool colors (light blue) representing low coupling scores and warm colors (red) representing high coupling scores. Coupling scores at or below the average scrambled value were assigned a score of zero and shaded dark blue (See Materials and Methods for details). (PNG) [file pone.0227668.s003.png]
