# Supplementary material for: Disease-relevant mutations alter amino acid co-evolution networks in the second nucleotide binding domain of CFTR
Source: PLoS One. 2020 Jan 24;15(1):e0227668. doi: 10.1371/journal.pone.0227668 (PMC6980524; doi:10.1371/journal.pone.0227668)

Amino acid frequencies in the >5,000 MSA

| Amino acid frequencies at | | | | |  |  | Amino acid frequencies | | | | |  |  | Amino acid frequencies | | | | |  |  |  |
| --- | --- | --- | --- | --- | --- | --- | --- | --- | --- | --- | --- | --- | --- | --- | --- | --- | --- | --- | --- | --- | --- |
|  |  |  |  |  |  |  | at position 1296 in full | | | | |  |  | at position 1358 in full | | | | |  |  |  |
| position 1303 in full MSA | | | | |  |  |  |  |  |  |  |  |  |  |  |  |  |  |  |  |  |
|  |  |  |  |  |  |  | MSA | | |  |  |  |  |  |  |  |  |  |  |  |  |
| - |  |  |  |  |  |  | - |  |  |  |  |  |  | - |  |  |  |  |  |  |  |
|  |  |  |  |  |  |  |  |  |  |  |  |  |  |  |  |  |  |  |  |  |  |
|  |  |  |  |  |  |  |  |  |  |  |  |  |  |  |  |  |  |  |  |  |  |
|  |  |  |  |  |  |  |  |  |  |  |  |  |  | Y |  |  |  |  |  |  |  |
| Y |  |  |  |  |  |  | Y |  |  |  |  |  |  |  |  |  |  |  |  |  |  |
|  |  |  |  |  |  |  |  |  |  |  |  |  |  | W |  |  |  |  |  |  |  |
| W |  |  |  |  |  |  | W |  |  |  |  |  |  |  |  |  |  |  |  |  |  |
|  |  |  |  |  |  |  |  |  |  |  |  |  |  | V |  |  |  |  |  |  |  |
| V |  |  |  |  |  |  | V |  |  |  |  |  |  |  |  |  |  |  |  |  |  |
|  |  |  |  |  |  |  |  |  |  |  |  |  |  | T |  |  |  |  |  |  |  |
| T |  |  |  |  |  |  | T |  |  |  |  |  |  |  |  |  |  |  |  |  |  |
|  |  |  |  |  |  |  |  |  |  |  |  |  |  | S |  |  |  |  |  |  |  |
| S |  |  |  |  |  |  | S |  |  |  |  |  |  |  |  |  |  |  |  |  |  |
|  |  |  |  |  |  |  |  |  |  |  |  |  |  | R |  |  |  |  |  |  |  |
| R |  |  |  |  |  |  | R |  |  |  |  |  |  |  |  |  |  |  |  |  |  |
|  |  |  |  |  |  |  |  |  |  |  |  |  |  | Q |  |  |  |  |  |  |  |
| Q |  |  |  |  |  |  | Q |  |  |  |  |  |  |  |  |  |  |  |  |  |  |
|  |  |  |  |  |  |  |  |  |  |  |  |  |  | P |  |  |  |  |  |  |  |
| P |  |  |  |  |  |  | P |  |  |  |  |  |  |  |  |  |  |  |  |  |  |
|  |  |  |  |  |  |  |  |  |  |  |  |  |  | N |  |  |  |  |  |  |  |
| N |  |  |  |  |  |  | N |  |  |  |  |  |  |  |  |  |  |  |  |  |  |
|  |  |  |  |  |  |  |  |  |  |  |  |  |  | M |  |  |  |  |  |  |  |
| M |  |  |  |  |  |  | M |  |  |  |  |  |  |  |  |  |  |  |  |  |  |
|  |  |  |  |  |  |  |  |  |  |  |  |  |  | L |  |  |  |  |  |  |  |
| L |  |  |  |  |  |  | L |  |  |  |  |  |  |  |  |  |  |  |  |  |  |
|  |  |  |  |  |  |  |  |  |  |  |  |  |  | K |  |  |  |  |  |  |  |
| K |  |  |  |  |  |  | K |  |  |  |  |  |  |  |  |  |  |  |  |  |  |
|  |  |  |  |  |  |  |  |  |  |  |  |  |  | I |  |  |  |  |  |  |  |
| I |  |  |  |  |  |  | I |  |  |  |  |  |  |  |  |  |  |  |  |  |  |
|  |  |  |  |  |  |  |  |  |  |  |  |  |  | H |  |  |  |  |  |  |  |
| H |  |  |  |  |  |  | H |  |  |  |  |  |  |  |  |  |  |  |  |  |  |
|  |  |  |  |  |  |  |  |  |  |  |  |  |  | G |  |  |  |  |  |  |  |
| G |  |  |  |  |  |  | G |  |  |  |  |  |  |  |  |  |  |  |  |  |  |
|  |  |  |  |  |  |  |  |  |  |  |  |  |  | F |  |  |  |  |  |  |  |
| F |  |  |  |  |  |  | F |  |  |  |  |  |  |  |  |  |  |  |  |  |  |
|  |  |  |  |  |  |  |  |  |  |  |  |  |  | E |  |  |  |  |  |  |  |
| E |  |  |  |  |  |  | E |  |  |  |  |  |  |  |  |  |  |  |  |  |  |
|  |  |  |  |  |  |  |  |  |  |  |  |  |  | D |  |  |  |  |  |  |  |
| D | |  |  |  |  |  | D |  |  |  |  |  |  |  |  |  |  |  |  |  |  |
|  |  |  |  |  |  |  |  |  |  |  |  |  |  | C |  |  |  |  |  |  |  |
|  |  |  |  |  |  |  | C |  |  |  |  |  |  |  |  |  |  |  |  |  |  |
| C | |  |  |  |  |  |  |  |  |  |  |  |  |  |  |  |  |  |  |  |  |
|  |  |  |  |  |  |  |  |  |  |  |  |  |  | A | |  |  |  |  |  |  |
|  |  |  |  |  |  |  | A | |  |  |  |  |  |  |  |  |  |  |  |  |  |
| A | |  |  |  |  |  |  |  |  |  |  |  |  |  |  |  |  |  |  |  |  |
|  |  |  |  |  |  |  |  |  |  |  |  |  |  |  |  |  |  |  |  |  |  |
|  |  |  |  |  |  |  |  |  |  |  |  |  |  | 0 | | | 0.5 | | 1 | |  |
| 0 | | | 0.5 | | 1 | | 0 | | | 0.5 | | 1 | |  |  |  |  |  |  |  |  |
|  |  |  |  |  |  |  |  |  |  |  |  |  |  |  |  |  |  |  |  |  |  |
|  |  |  |  |  |  |  |  |  |  |  |  |  |  |  |  |  |  |  |  |  |  |


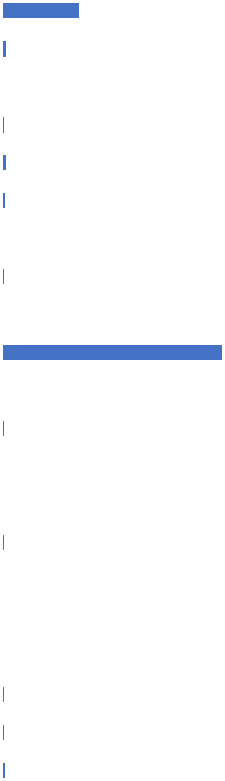

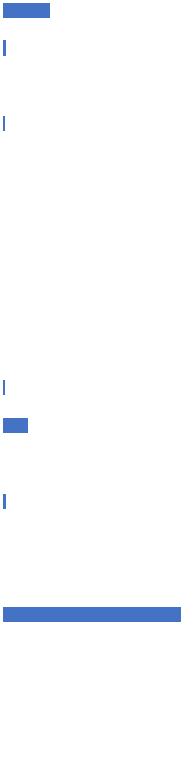

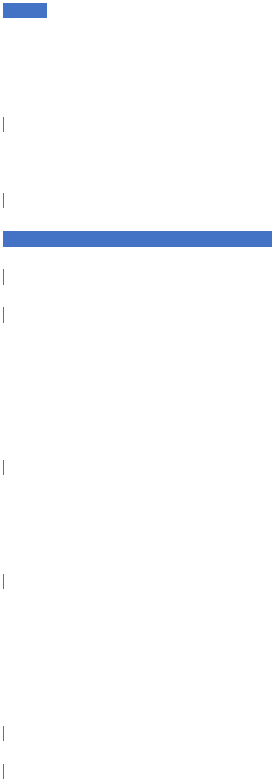


Amino Acid Frequencies at Position 1296


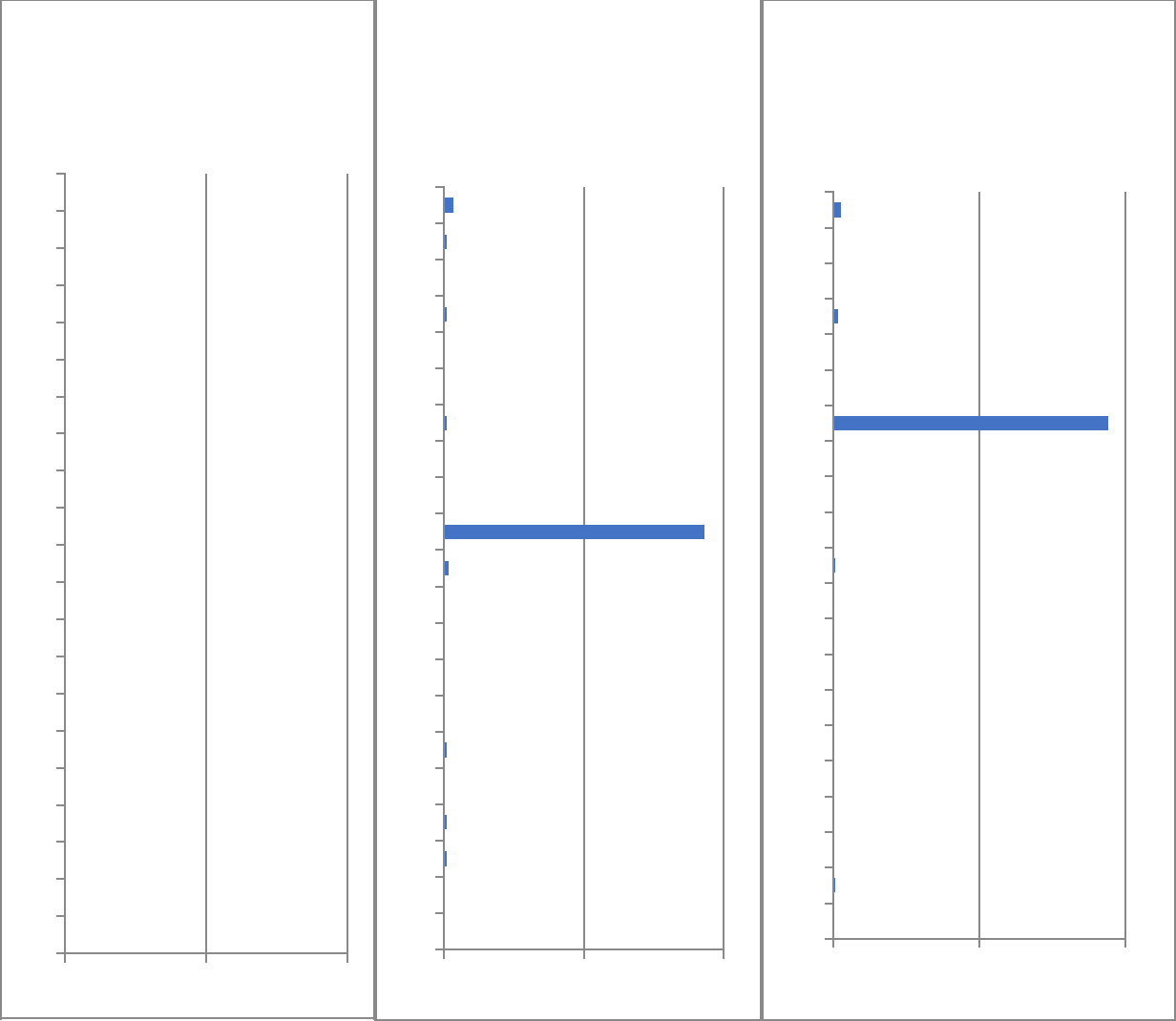


Amino acid frequency at position 1358 in subset

1296I.

-

Y

W

V

T

S

R

Q

P

N

M

L

K

I

H

G

F

E

D

C

A

0 0.5 1

Amino acid frequency at

positions 1303 in subset

1296I.

-

Y

W

V

T

S

R

Q

P

N

M

L

K

I

H

G

F

E

D

C

A

0 0.5 1

Amino acid frequencies at position 1358 in subset 1296Y

-

Y

W

V

T

S

R

Q

P

N

M

L

K

I

H

G

F

E

D

C

A

0 0.5 1

Amino acid frequencies at position 1303 in subset 1296Y

-

W

T

R

P

M

K

H

F

D

A

Amino acid frequencies at position 1358 in subset 1296L


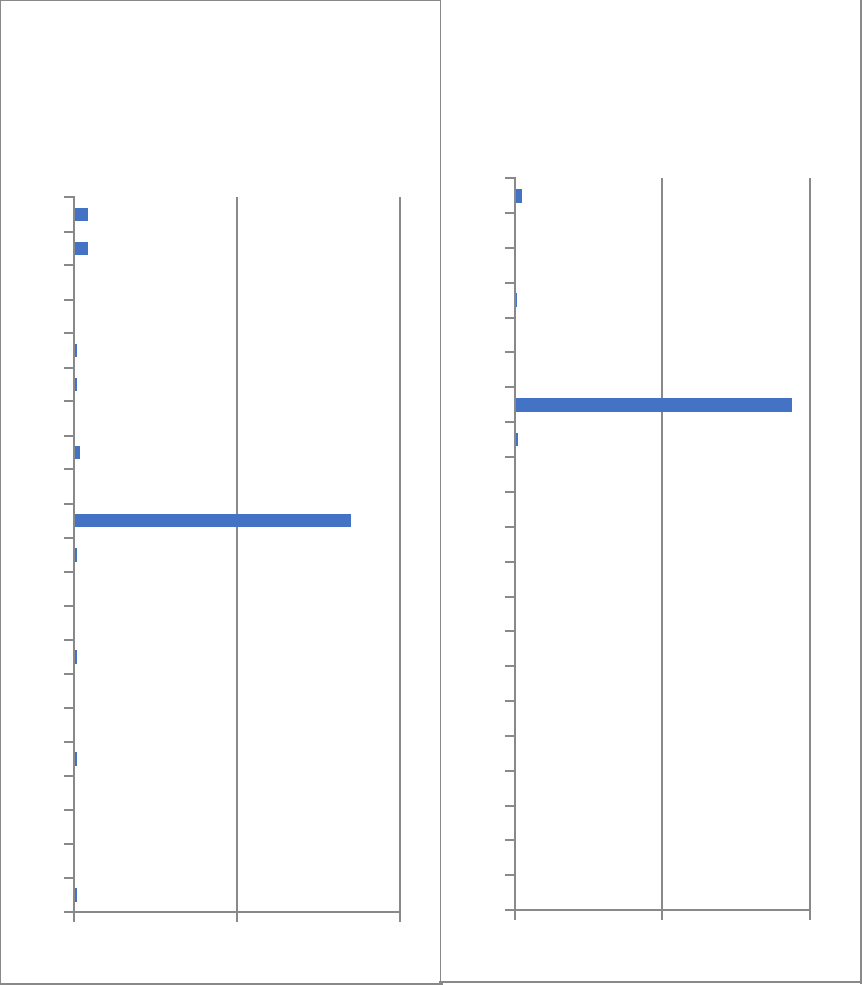


-

W

T

R

P

M

K

H

F

D

A

Amino acid frequencies at position 1303 in subset 1296L


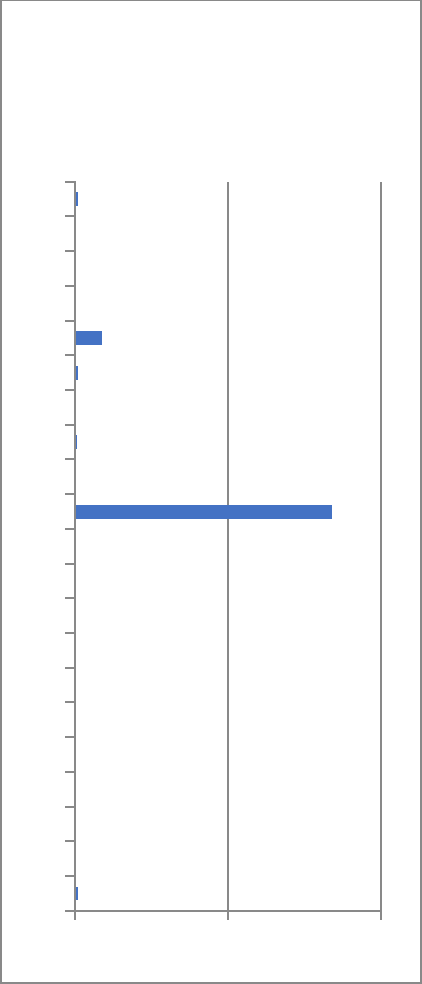


-

W

T

R

P

M

K

H

F

D

A

| 0 | 0.5 | 1 | 0 | 0.5 | 1 |
| --- | --- | --- | --- | --- | --- |

0 0.5 1

Amino Acid Frequencies at the position 1303

| Amino acid | | | | |  |  | Amino acid | | | | |  |  |  | Amino acid | | | |  |  | Amino acid | | | |  |  |  |
| --- | --- | --- | --- | --- | --- | --- | --- | --- | --- | --- | --- | --- | --- | --- | --- | --- | --- | --- | --- | --- | --- | --- | --- | --- | --- | --- | --- |
|  |  |  |  |  |  |  | frequencies at | | | | |  |  |  | frequencies at | | | |  |  | frequencies at | | | |  |  |  |
| frequencies at | | | | |  |  |  |  |  |  |  |  |  |  |  |  |  |  |  |  |  |  |  |  |  |  |  |
|  |  |  |  |  |  |  | position 1358 in | | | | |  |  |  | position 1296 in | | | |  |  | position 1358 in | | | |  |  |  |
| position 1296 in | | | | |  |  |  |  |  |  |  |  |  |  |  |  |  |  |  |  |  |  |  |  |  |  |  |
|  |  |  |  |  |  |  | 1303Y subset. | | | | |  |  |  |  | 1303T subset. | | |  |  | 1303T subset. | | | |  |  |  |
| 1303Y subset. | | | | |  |  |  |  |  |  |  |  |  | - |  |  |  |  |  |  |  |  |  |  |  |  |  |
|  |  |  |  |  |  |  |  |  |  |  |  |  |  |  |  |  |  |  |  |  |  |  |  |  |  |  |  |
|  |  |  |  |  |  |  |  |  |  |  |  |  |  |  |  |  |  |  |  |  | - |  |  |  |  |  |  |
| - |  |  |  |  |  |  | - |  |  |  |  |  |  | Y |  |  |  |  |  |  | Y |  |  |  |  |  |  |
| W |  |  |  |  |  |  | W |  |  |  |  |  |  | W |  |  |  |  |  |  | W |  |  |  |  |  |  |
|  |  |  |  |  |  |  |  |  |  |  |  |  |  | V |  |  |  |  |  |  | V |  |  |  |  |  |  |
| T |  |  |  |  |  |  | T |  |  |  |  |  |  | T |  |  |  |  |  |  | T |  |  |  |  |  |  |
|  |  |  |  |  |  |  |  |  |  |  |  |  |  | S |  |  |  |  |  |  | S |  |  |  |  |  |  |
|  |  |  |  |  |  |  |  |  |  |  |  |  |  |  |  |  |  |  |  |  |  |  |  |  |  |  |  |
| R |  |  |  |  |  |  | R |  |  |  |  |  |  | R |  |  |  |  |  |  | R |  |  |  |  |  |  |
|  |  |  |  |  |  |  |  |  |  |  |  |  |  |  |  |  |  |  |  |  |  |  |  |  |  |  |  |
|  |  |  |  |  |  |  |  |  |  |  |  |  |  | Q |  |  |  |  |  |  | Q |  |  |  |  |  |  |
|  |  |  |  |  |  |  |  |  |  |  |  |  |  |  |  |  |  |  |  |  |  |  |  |  |  |  |  |
|  |  |  |  |  |  |  |  |  |  |  |  |  |  |  |  |  |  |  |  |  |  |  |  |  |  |  |  |
|  |  |  |  |  |  |  |  |  |  |  |  |  |  | P |  |  |  |  |  |  | P |  |  |  |  |  |  |
| P | |  |  |  |  |  | P | |  |  |  |  |  |  |  |  |  |  |  |  |  |  |  |  |  |  |  |
|  |  |  |  |  |  |  |  |  |  |  |  |  |  | N |  |  |  |  |  |  | N | | |  |  |  |  |
|  |  |  |  |  |  |  |  |  |  |  |  |  |  |  |  |  |  |  |  |  |  |  |  |  |  |  |  |
|  |  |  |  |  |  |  |  |  |  |  |  |  |  | M |  |  |  |  |  |  | M |  |  |  |  |  |  |
| M | |  |  |  |  |  | M | |  |  |  |  |  |  |  |  |  |  |  |  |  |  |  |  |  |  |  |
|  |  |  |  |  |  |  |  |  |  |  |  |  |  | L |  |  |  |  |  |  | L | | |  |  |  |  |
|  |  |  |  |  |  |  |  |  |  |  |  |  |  |  |  |  |  |  |  |  |  |  |  |  |  |  |  |
|  |  |  |  |  |  |  |  |  |  |  |  |  |  | K |  |  |  |  |  |  | K |  |  |  |  |  |  |
| K | |  |  |  |  |  | K | |  |  |  |  |  |  |  |  |  |  |  |  |  |  |  |  |  |  |  |
|  |  |  |  |  |  |  |  |  |  |  |  |  |  | I |  |  |  |  |  |  | I | | |  |  |  |  |
|  |  |  |  |  |  |  |  |  |  |  |  |  |  |  |  |  |  |  |  |  |  |  |  |  |  |  |  |
| H |  |  |  |  |  |  | H |  |  |  |  |  |  | H |  |  |  |  |  |  | H |  |  |  |  |  |  |
|  |  |  |  |  |  |  |  |  |  |  |  |  |  |  |  |  |  |  |  |  |  |  |  |  |  |  |  |
|  |  |  |  |  |  |  |  |  |  |  |  |  |  | G |  |  |  |  |  |  | G | | |  |  |  |  |
|  |  |  |  |  |  |  |  |  |  |  |  |  |  |  |  |  |  |  |  |  |  |  |  |  |  |  |  |
| F |  |  |  |  |  |  | F |  |  |  |  |  |  | F |  |  |  |  |  |  | F |  |  |  |  |  |  |
|  |  |  |  |  |  |  |  |  |  |  |  |  |  |  |  |  |  |  |  |  |  |  |  |  |  |  |  |
|  |  |  |  |  |  |  |  |  |  |  |  |  |  | E |  |  |  |  |  |  | E |  |  |  |  |  |  |
| D |  |  |  |  |  |  | D |  |  |  |  |  |  | D |  |  |  |  |  |  | D |  |  |  |  |  |  |
| A |  |  |  |  |  |  |  |  |  |  |  |  |  | C |  |  |  |  |  |  | C |  |  |  |  |  |  |
|  |  |  |  |  |  |  | A |  |  |  |  |  |  | A |  |  |  |  |  |  | A |  |  |  |  |  |  |
|  |  |  |  |  |  |  |  |  |  |  |  |  |  |  |  |  |  |  |  |  |  |  |  |  |  |  |  |
| 0 | | | 0.5 | | 1 | | 0 | | | 0.5 | | 1 | |  | 0 | | | 0.5 | 1 | | 0 | | | 1 | 2 | |  |
|  |  |  |  |  |  |  |  |  |  |  |  |  |  |  |  |  |  |  |  |  |  |  |  |  |  |  |  |


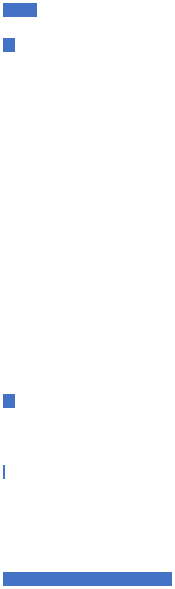

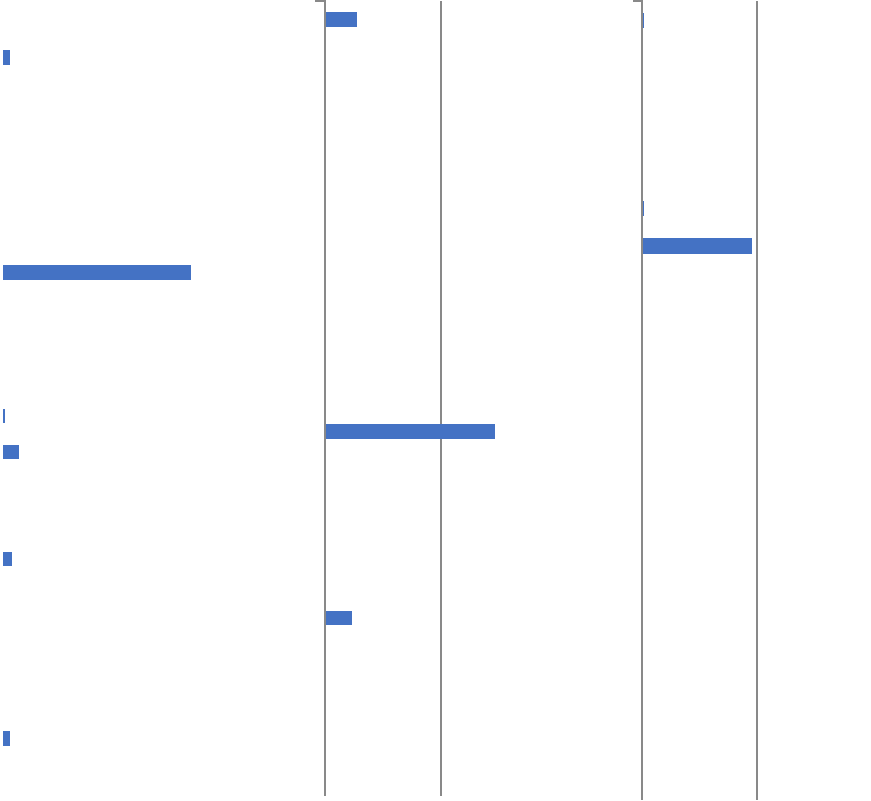

Supplement: S5 Fig — Amino acid frequency distributions at position 1296 (site 1 –Szollosi [15]), 1303 (site 2 –Szollosi [15]), and 1358 (site 3 –Szollosi [15]) in full (wildtype) MSA. Amino acid frequency distributions at position 1303 in 1296 (1296I, 1296Y, 1296L) and 1358 (1296I, 1296Y, 1296L) perturbations. (DOCX) [file pone.0227668.s005.docx]
