# Supplementary material for: Disease-relevant mutations alter amino acid co-evolution networks in the second nucleotide binding domain of CFTR
Source: PLoS One. 2020 Jan 24;15(1):e0227668. doi: 10.1371/journal.pone.0227668 (PMC6980524; doi:10.1371/journal.pone.0227668)

|  | 1296 in >50,000 |  |  |  | 1303 in >50,000 | | |  |  | 1296 in 1303Q | | | | | | 1303 in 1296S | | | | | |  |
| --- | --- | --- | --- | --- | --- | --- | --- | --- | --- | --- | --- | --- | --- | --- | --- | --- | --- | --- | --- | --- | --- | --- |
|  | MSA |  |  |  |  | MSA | |  |  |  |  | Subset | |  |  |  |  | Subset | |  |  |  |
| - |  |  |  | - |  |  |  |  |  | - |  |  |  |  |  | - |  |  |  |  |  |  |
|  |  |  |  |  |  |  |  |  |  |  |  |  |  |  |  |  |  |  |  |  |  |  |
| Y |  |  |  | Y |  |  |  |  |  | Y |  |  |  |  |  | Y |  |  |  |  |  |  |
| W |  |  |  | W |  |  |  |  |  | W |  |  |  |  |  | W |  |  |  |  |  |  |
| V |  |  |  | V |  |  |  |  |  | V |  |  |  |  |  | V |  |  |  |  |  |  |
| T |  |  |  | T |  |  |  |  |  | T |  |  |  |  |  | T |  |  |  |  |  |  |
| S |  |  |  | S |  |  |  |  |  | S |  |  |  |  |  | S |  |  |  |  |  |  |
| R |  |  |  | R |  |  |  |  |  | R |  |  |  |  |  | R |  |  |  |  |  |  |
| Q |  |  |  | Q |  |  |  |  |  | Q |  |  |  |  |  | Q |  |  |  |  |  |  |
| P |  |  |  | P |  |  |  |  |  | P |  |  |  |  |  | P |  |  |  |  |  |  |
| N |  |  |  | N |  |  |  |  |  | N |  |  |  |  |  | N |  |  |  |  |  |  |
| M |  |  |  | M |  |  |  |  |  | M |  |  |  |  |  | M |  |  |  |  |  |  |
| L |  |  |  | L |  |  |  |  |  | L |  |  |  |  |  | L |  |  |  |  |  |  |
| K |  |  |  | K |  |  |  |  |  | K |  |  |  |  |  | K |  |  |  |  |  |  |
| I |  |  |  | I |  |  |  |  |  | I |  |  |  |  |  | I |  |  |  |  |  |  |
| H |  |  |  | H |  |  |  |  |  | H |  |  |  |  |  | H |  |  |  |  |  |  |
| G |  |  |  | G |  |  |  |  |  | G |  |  |  |  |  | G |  |  |  |  |  |  |
| F |  |  |  | F |  |  |  |  |  | F |  |  |  |  |  | F |  |  |  |  |  |  |
| E |  |  |  | E |  |  |  |  |  | E |  |  |  |  |  | E |  |  |  |  |  |  |
| D |  |  |  | D |  |  |  |  |  | D |  |  |  |  |  | D |  |  |  |  |  |  |
| C |  |  |  | C |  |  |  |  |  | C |  |  |  |  |  | C |  |  |  |  |  |  |
| A |  |  |  | A |  |  |  |  |  | A |  |  |  |  |  | A |  |  |  |  |  |  |
| 0 | | 0.5 | | 0 | | 0.5 | | 1 | | 0 | | 0.5 | | 1 | | 0 | | 0.2 | | 0.4 | |  |
|  |  |  |  |  |  |  |  |  |  |  |  |  |  |  |  |  |  |  |  |  |  |  |


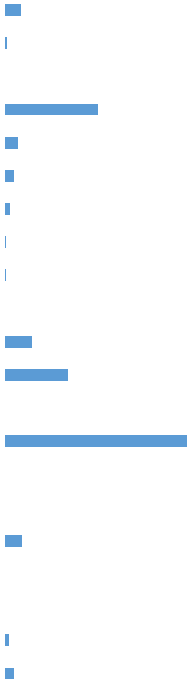

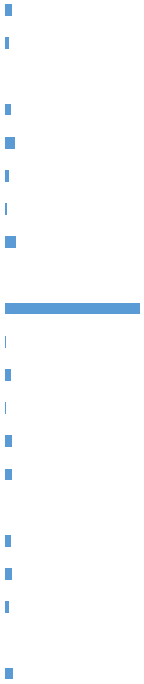

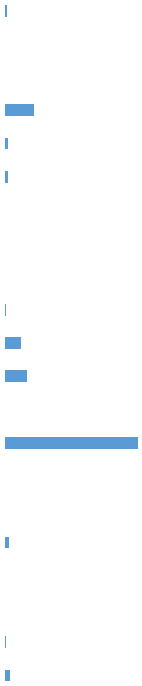

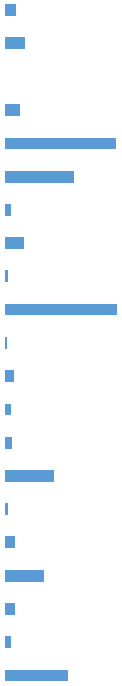


|  |  | 1358 in >50,000 | |  |  |  |  | 1358 in | | 1296 in 1358A | | | | | |  |
| --- | --- | --- | --- | --- | --- | --- | --- | --- | --- | --- | --- | --- | --- | --- | --- | --- |
|  |  | MSA | |  |  | 1303Q Subset | | | |  |  | Subset | |  |  |  |
| - |  |  |  |  |  | - |  |  |  | - |  |  |  |  |  |  |
|  |  |  |  |  |  |  |  |  |  |  |  |  |  |  |  |  |
| Y |  |  |  |  |  | Y |  |  |  | Y |  |  |  |  |  |  |
| W |  |  |  |  |  | W |  |  |  | W |  |  |  |  |  |  |
| V |  |  |  |  |  | V |  |  |  | V |  |  |  |  |  |  |
| T |  |  |  |  |  | T |  |  |  | T |  |  |  |  |  |  |
| S |  |  |  |  |  | S |  |  |  | S |  |  |  |  |  |  |
| R |  |  |  |  |  | R |  |  |  | R |  |  |  |  |  |  |
| Q |  |  |  |  |  | Q |  |  |  | Q |  |  |  |  |  |  |
| P |  |  |  |  |  | P |  |  |  | P |  |  |  |  |  |  |
| N |  |  |  |  |  | N |  |  |  | N |  |  |  |  |  |  |
| M |  |  |  |  |  | M |  |  |  | M |  |  |  |  |  |  |
| L |  |  |  |  |  | L |  |  |  | L |  |  |  |  |  |  |
| K |  |  |  |  |  | K |  |  |  | K |  |  |  |  |  |  |
| I |  |  |  |  |  | I |  |  |  | I |  |  |  |  |  |  |
| H |  |  |  |  |  | H |  |  |  | H |  |  |  |  |  |  |
| G |  |  |  |  |  | G |  |  |  | G |  |  |  |  |  |  |
| F |  |  |  |  |  | F |  |  |  | F |  |  |  |  |  |  |
| E |  |  |  |  |  | E |  |  |  | E |  |  |  |  |  |  |
| D |  |  |  |  |  | D |  |  |  | D |  |  |  |  |  |  |
| C |  |  |  |  |  | C |  |  |  | C |  |  |  |  |  |  |
| A |  |  |  |  |  | A |  |  |  | A |  |  |  |  |  |  |
| 0 | | 0.5 | | 1 | | 0 | | 0.5 | | 0 | | 0.2 | | 0.4 | |  |
|  |  |  |  |  |  |  |  |  |  |  |  |  |  |  |  |  |


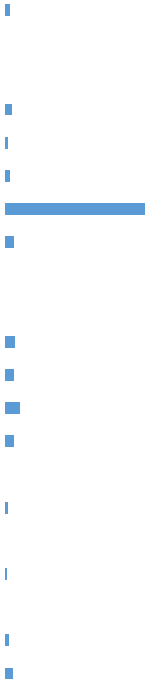

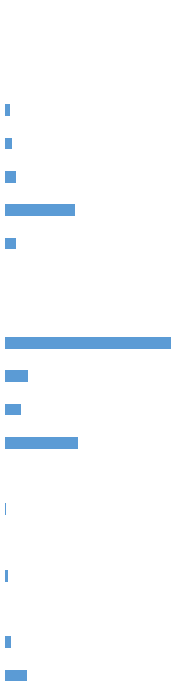

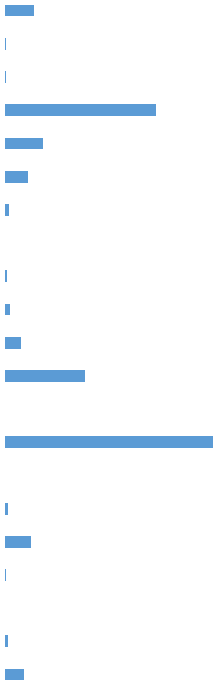

Supplement: S6 Fig — Amino acid frequency distributions at position 1296 (site 1 –Szollosi [15]) and 1303 (site 2 –Szollosi [15]) in full (wildtype) MSA. Amino acid frequency distributions at position 1296 in 1303 perturbations (1303Q, 1358A). Amino acid frequency distributions at position 1303 in 1296S perturbation and 1358 in 1303Q perturbation. (DOCX) [file pone.0227668.s006.docx]
