# Supplementary material for: Disease-relevant mutations alter amino acid co-evolution networks in the second nucleotide binding domain of CFTR
Source: PLoS One. 2020 Jan 24;15(1):e0227668. doi: 10.1371/journal.pone.0227668 (PMC6980524; doi:10.1371/journal.pone.0227668)
Supplement: S7 Fig — (DOCX) [file pone.0227668.s007.docx]

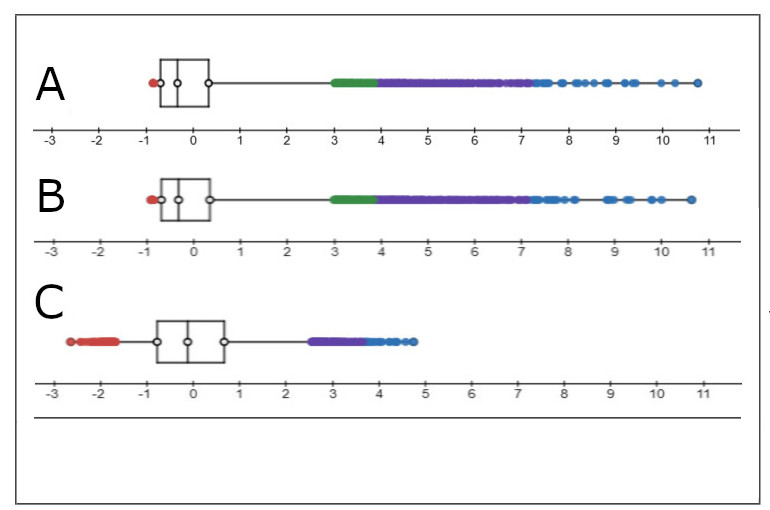

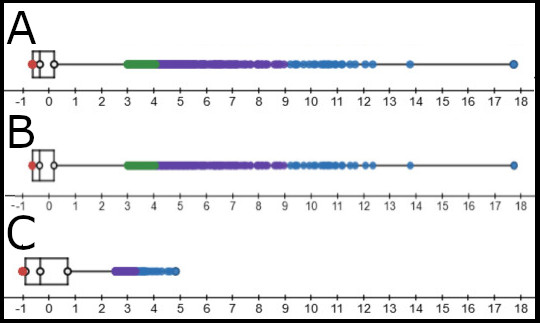

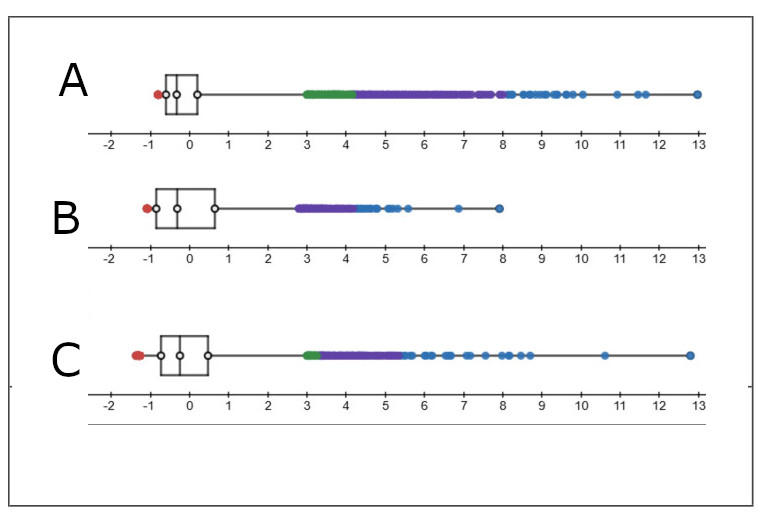


ELSC

OMES

McBASC

FULL (Wildtype) MSA

Fig. S7A: Boxplots of the Z-scores calculated from the distribution of the co-evolution scores obtained from Full (Wildtype) MSA for ELSC, OMES, and McBASC algorithms. The blue dots are the top 25, purple is the next top 250, red is the bottom 275, and green are the outliers (-/+ 3 standard deviations) not included in either of the other colors. Boxes represent the 25th and 75th percentile and the middle line represents the median. A) Original MSA B) Randomized MSA C) Scrambled MSA

S1235R

ELSC


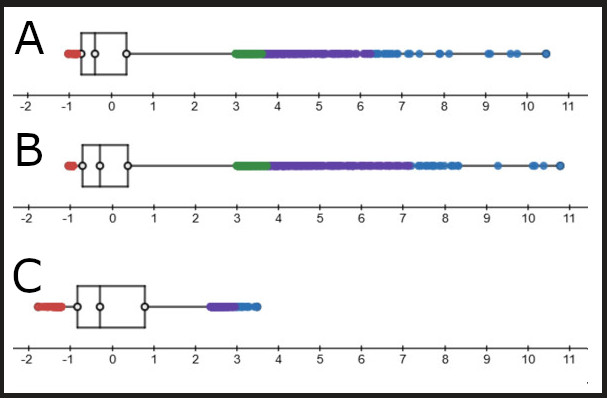


OMES


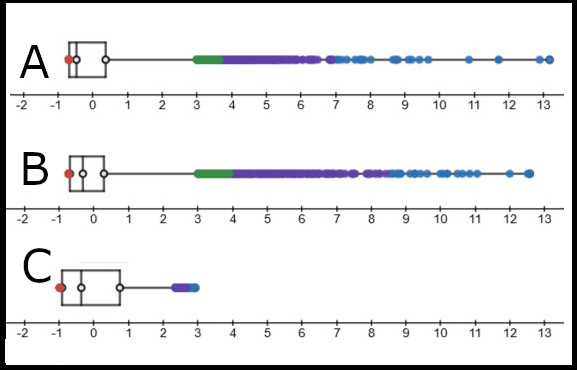


McBASC


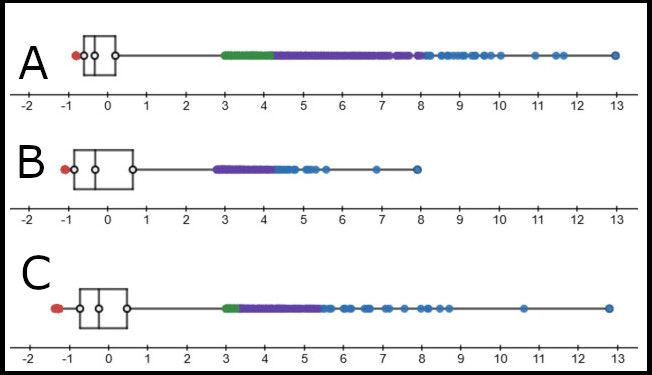


Fig. S7B: Boxplots of the Z-scores calculated from the distribution of the co-evolution scores obtained from S1235R MSA for ELSC, OMES, and McBASC algorithms. The blue dots are the top 25, purple is the next top 250, red is the bottom 275, and green are the outliers (-/+ 3 standard deviations) not included in either of the other colors. Boxes represent the 25th and 75th percentile and the middle line represents the median. A) Original MSA B) Randomized MSA C) Scrambled MSA

S1251T

ELSC


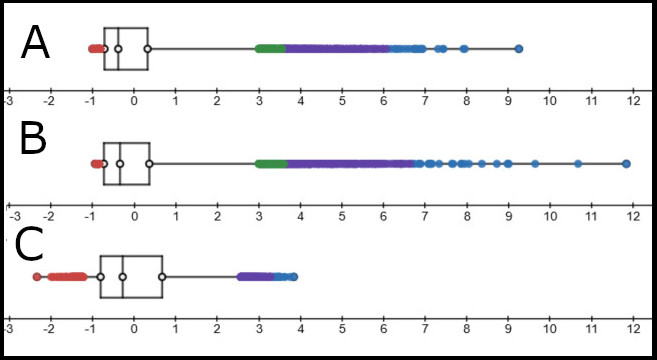


OMES


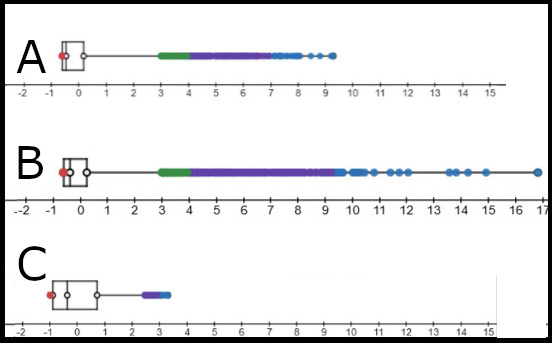


McBASC


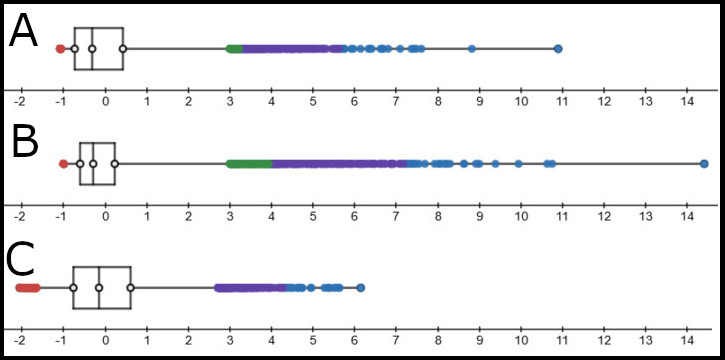


Fig. S7C: Boxplots of the Z-scores calculated from the distribution of the co-evolution scores obtained from S1251T MSA for ELSC, OMES, and McBASC algorithms. The blue dots are the top 25, purple is the next top 250, red is the bottom 275, and green are the outliers (-/+ 3 standard deviations) not included in either of the other colors. Boxes represent the 25th and 75th percentile and the middle line represents the median. A) Original MSA B) Randomized MSA C) Scrambled MSA

N1303T

ELSC


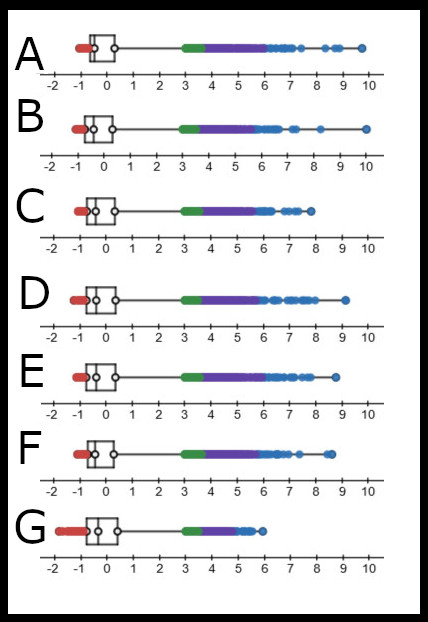


OMES


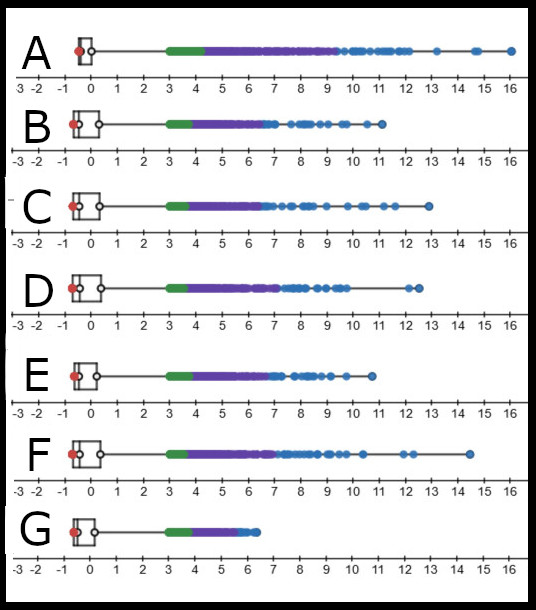


McBASC


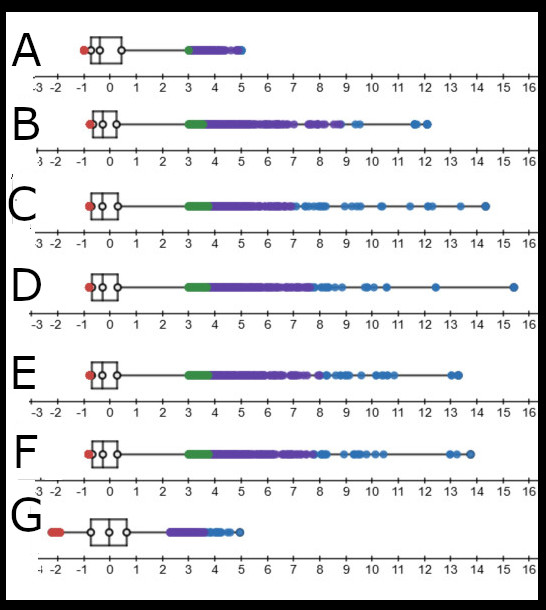


Fig. S7D: Boxplots of the Z-scores calculated from the distribution of the co-evolution scores obtained from N1303T MSA for ELSC, OMES, and McBASC algorithms. The blue dots are the top 25, purple is the next top 250, red is the bottom 275, and green are the outliers (-/+ 3 standard deviations) not included in either of the other colors. Boxes represent the 25th and 75th percentile and the middle line represents the median. A) Original MSA B-F) Randomized MSA G) Scrambled MSA
