# Supplementary figures and images for: Correction: Disease-relevant mutations alter amino acid co-evolution networks in the second nucleotide binding domain of CFTR
Source: PLoS One. 2020 Feb 27;15(2):e0229986. doi: 10.1371/journal.pone.0229986 (PMC7046266; doi:10.1371/journal.pone.0229986)

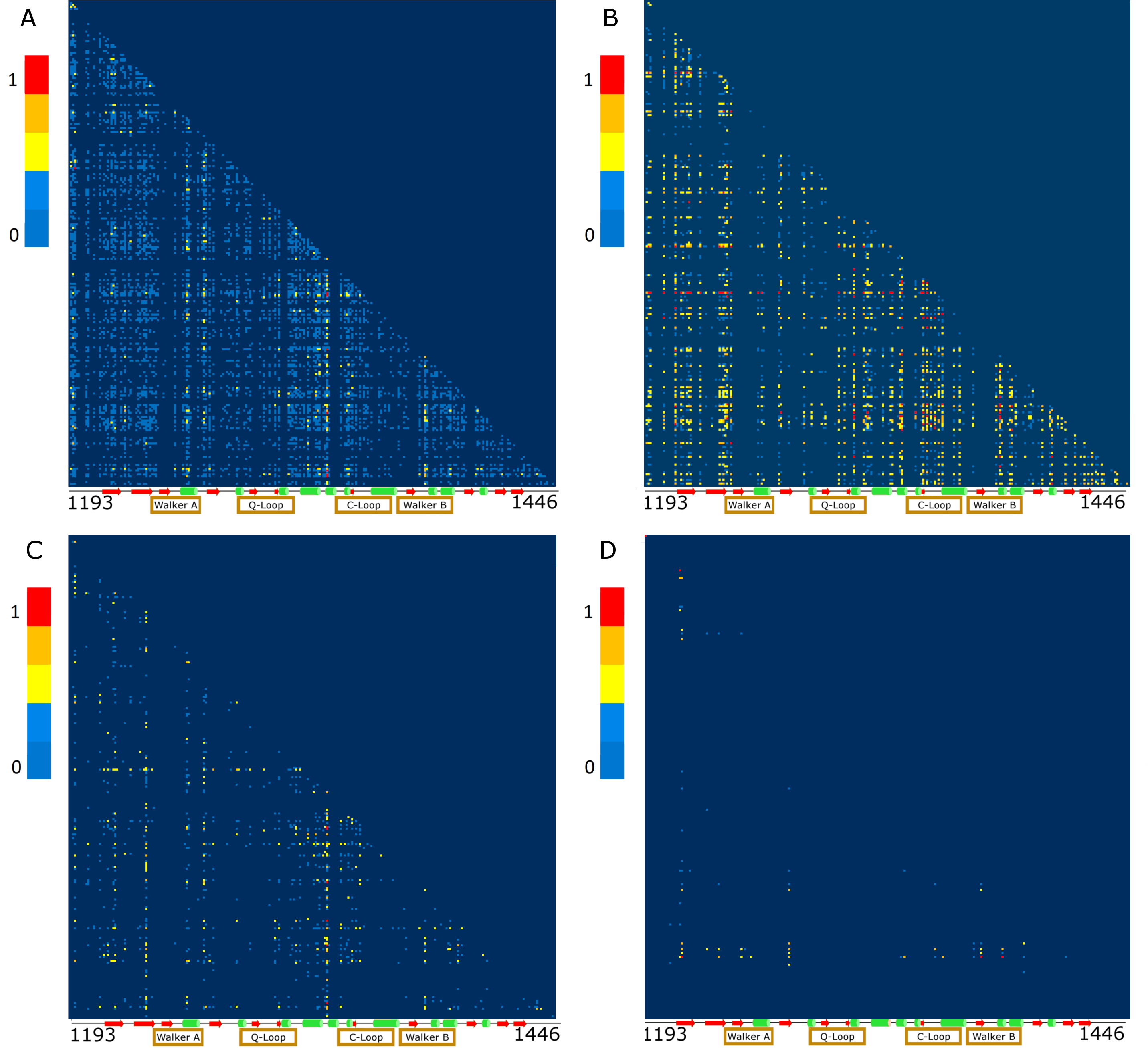

Supplement: S1 Fig — Heat maps represent coupled positions identified by the Statistical Coupling Analysis for perturbations (A) Ser1251, (B) Ser1235, (C) Asn1303, and (D) for the wildtype (Full MSA). High scores are represented by warm colors (red), and cool colors depict low scores. Coupled positions at or below the scrambled score for that MSA were colored dark blue. (PNG) [file pone.0229986.s001.png]
